# The expression of aminoglycoside resistance genes in integron cassettes is not controlled by riboswitches

**DOI:** 10.1093/nar/gkac662

**Published:** 2022-08-10

**Authors:** Alberto Hipólito, Lucía García-Pastor, Paula Blanco, Filipa Trigo da Roza, Nicolas Kieffer, Ester Vergara, Thomas Jové, Julio Álvarez, José Antonio Escudero

**Affiliations:** Departamento de Sanidad Animal, Facultad de Veterinaria de la Universidad Complutense de Madrid, Spain; VISAVET Health Surveillance Centre, Universidad Complutense de Madrid, Spain; Departamento de Sanidad Animal, Facultad de Veterinaria de la Universidad Complutense de Madrid, Spain; VISAVET Health Surveillance Centre, Universidad Complutense de Madrid, Spain; Departamento de Sanidad Animal, Facultad de Veterinaria de la Universidad Complutense de Madrid, Spain; VISAVET Health Surveillance Centre, Universidad Complutense de Madrid, Spain; Departamento de Sanidad Animal, Facultad de Veterinaria de la Universidad Complutense de Madrid, Spain; VISAVET Health Surveillance Centre, Universidad Complutense de Madrid, Spain; Departamento de Sanidad Animal, Facultad de Veterinaria de la Universidad Complutense de Madrid, Spain; VISAVET Health Surveillance Centre, Universidad Complutense de Madrid, Spain; Departamento de Sanidad Animal, Facultad de Veterinaria de la Universidad Complutense de Madrid, Spain; VISAVET Health Surveillance Centre, Universidad Complutense de Madrid, Spain; INSERM, CHU Limoges, RESINFIT, University of Limoges, Limoges, France; Departamento de Sanidad Animal, Facultad de Veterinaria de la Universidad Complutense de Madrid, Spain; VISAVET Health Surveillance Centre, Universidad Complutense de Madrid, Spain; Departamento de Sanidad Animal, Facultad de Veterinaria de la Universidad Complutense de Madrid, Spain; VISAVET Health Surveillance Centre, Universidad Complutense de Madrid, Spain

## Abstract

Regulation of gene expression is a key factor influencing the success of antimicrobial resistance determinants. A variety of determinants conferring resistance against aminoglycosides (Ag) are commonly found in clinically relevant bacteria, but whether their expression is regulated or not is controversial. The expression of several Ag resistance genes has been reported to be controlled by a riboswitch mechanism encoded in a conserved sequence. Yet this sequence corresponds to the integration site of an integron, a genetic platform that recruits genes of different functions, making the presence of such a riboswitch counterintuitive. We provide, for the first time, experimental evidence against the existence of such Ag-sensing riboswitch. We first tried to reproduce the induction of the well characterized *aacA5* gene using its native genetic environment, but were unsuccessful. We then broadened our approach and analyzed the inducibility of all AgR genes encoded in integrons against a variety of antibiotics. We could not observe biologically relevant induction rates for any gene in the presence of several aminoglycosides. Instead, unrelated antibiotics produced mild but consistently higher increases in expression, that were the result of pleiotropic effects. Our findings rule out the riboswitch control of aminoglycoside resistance genes in integrons.

## INTRODUCTION

The expression of antibiotic resistance (AR) genes can influence the phenotype and the fitness of the bacterial host, and is a fundamental factor driving their fate in the clinical setting (1). Inducible resistance mechanisms can alleviate the cost of resistance in the absence of antibiotics while preserving a high resistance phenotype in its presence (2–4). Several mechanisms are known to induce the expression of AR genes in the presence of antibiotics, like ribosome stalling (5,6), ribosome-mediated transcriptional attenuation (7,8), and the sensing of the antibiotic molecules (9) or their effects (10,11). Because the cost of resistance is the main driver of its reversibility at the community level (12), a clear understanding of the transcriptional and translational mechanisms regulating the expression of resistance genes is key in the fight against AR.

Aminoglycosides (Ag) are critically important antibiotics (13) against which the most common resistance mechanism is enzymatic modification. Many such modifying enzymes are plasmid-borne and circulate among clinically relevant species. To fight against the emergence and spread of Ag resistant bacteria it is necessary to understand the expression of these determinants. Notably, the expression of AgR genes has recently been claimed to be under the control of a riboswitch (14). The authors observed a strong level of conservation of the 5′ leader RNA of several AgR genes and suspected a common regulation mechanism. They investigated experimentally the (6′)-*N*-acetyltransferase encoded in *aacA5* (Genbank L06163) and found a potential secondary structure in the 5′ untranslated region (UTR) of its mRNA that involved a large part of the conserved region. Through a series of experiments combining bioinformatic, genetic and biochemical techniques, they concluded that 4–6 disubstituted deoxystreptamine (4–6-DDs) aminoglycosides bind to this structure and destabilize it, releasing the trapped Shine-Dalgarno (SD) sequence and allowing for the translation of the gene. This structure would hence act as a riboswitch. Given the sequence conservation they observed among AgR genes, in follow up works from the same group, they investigated the regulation of other AgR genes using a similar methodology and concluded that riboswitches are also present in other acetyltransferases (15) and adenyltransferases (16).

**Figure 1.**
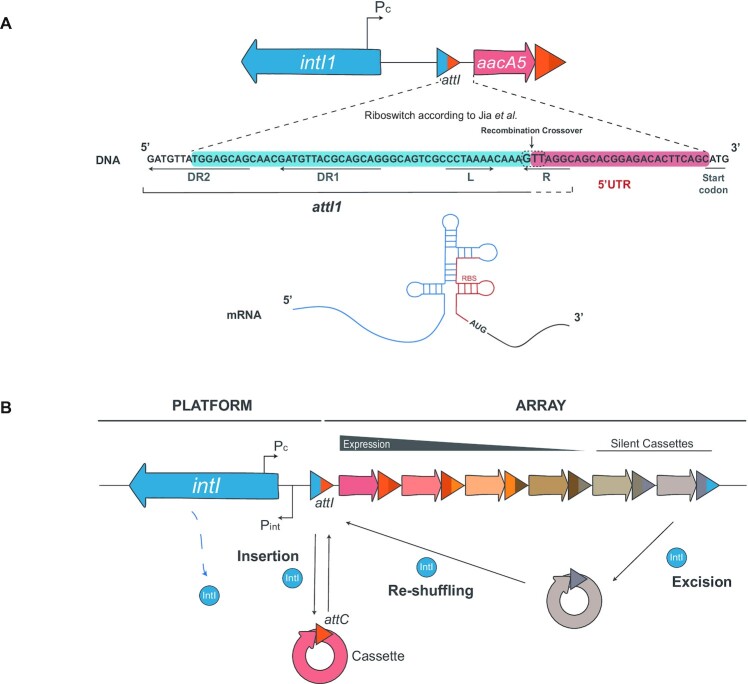
The aminoglycoside sensing riboswitch described in an integron cassette is counterintuitive. (**A**) Diagram of the riboswitch described in (14) upstream the *aacA5* integron cassette. A large part of the structure is composed of the *attI1* integration site of class 1 integrons (blue shade) including the direct repeats (DR) 1 and 2 (partially) and the L box. This region is the sequence found by the authors to be conserved upstream aminoglycoside resistance genes. Cassettes start at the crossover point between the G and the T of the conserved 5′-GTT-3′ triplet (dotted box). It is of note that most of the R box (5′-GTTRRRY-3′) in *attI* sites is provided by cassettes in first position, but are a functional part of the site. (**B**) Schematic representation of an integron. The conserved part (the platform) includes the integrase coding gene, the Pc and Pint promoters and the *attI* integration site. Integrases insert cassettes in *attI* -where they are expressed from the Pc promoter- and form an array of variable content. They can also shuffle the order of cassettes through excision and integration reactions, modulating their expression.

A puzzling aspect of these riboswitch-controlled genes is that they are encoded in integron cassettes, and that the conserved region encoding a large part of the riboswitch is actually the integration site (*attI*) of a class 1 integron, where genes of unrelated functions are inserted (Figure 1A) (17). Integrons are bacterial recombination platforms that recruit new genes encoded in mobile genetic elements called cassettes (Figure 1B) (18,19) (reviewed in (20,21)). They do so through site-specific DNA-recombination reactions between the *attC* site in cassettes and the *attI* site within the integron platform (22–24). Sequential integrations at *attI* lead to the formation of a cassette array of variable content that is expressed from the P_C_ promoter located immediately upstream the *attI* site (25). Hence, cassettes acquired recently are closer to the P_C_ and therefore more expressed than those acquired earlier. Yet, the order of cassettes in an array is dynamic: under stress conditions or during horizontal gene transfer, integrons can reshuffle them to modulate their expression (26–29) (Figure 1B). Integrons are ancient structures found in ∼10% of the genomes in the databases (30,31). Five integron classes carrying AR cassettes have been transposed onto plasmids and have reached our hospitals. These elements are called *mobile integrons* (MIs), in contrast to the native *sedentary chromosomal integrons* (SCIs) in environmental bacteria. MIs are nowadays commonplace in Gram negative clinical isolates carrying small subsets of typically 1 to 6 resistance cassettes against virtually all antibiotic families. The class 1 integron is by far the most prevalent and important MI (32) and is the platform in which the riboswitch was described.

The variety of functions encoded in integron cassettes makes the presence of such a riboswitch counterintuitive, since the expression of unrelated genes would be contingent on the presence of aminoglycosides. Also, in the initial report on these putative riboswitches, the induction levels observed for *aacA5* were arguably very low (1.5–2.5-fold) and it has been suggested that these effects might be the result of pleiotropic effects of antibiotics on protein production, rather than the consequence of a specific induction mechanism (33). Yet, data supporting this claim was not provided and is still nowadays unavailable. Hence the presence of Ag riboswitches in integron AgR cassettes remains today counterintuitive and controversial, and experimental proof from independent laboratories is necessary. In this work we provide, for the first time, experimental evidence addressing the validity of the Ag-sensing riboswitch. We have recreated some of the experiments for *aacA5* mimicking the native genetic environment of cassettes in a class 1 integron, but could not observe an induction by aminoglycosides. We have then extended our approach to all AgR cassettes found in integrons (64), to provide an exhaustive analysis on the inducibility of these elements. Our data reveal that AgR genes in cassettes are not repressed in the absence of Ag, nor induced in their presence. We show that some unrelated antibiotics can produce higher (although still mild) variations in expression levels in AgR cassettes, and we prove that these are the result of pleiotropic effects of antibiotics. Altogether our data rule out the presence of a riboswitch controlling aminoglycoside resistance genes in integron cassettes.

## MATERIALS AND METHODS

### Bacterial Strains, Antibiotics and Culture Conditions

*Escherichia coli* MG1655 was used as recipient for all plasmid constructions in this work. Bacterial strains (Supplementary Table S2) were grown at 37°C in Müeller Hinton (MH; Oxoid, UK), lysogeny broth (LB) or LB agar (1.5%) (BD, France). Zeocin was added at 100 μg/ml to maintain pMBA and pMBA-derived plasmids in *E. coli*. Liquid cultures were incubated in an Infors Multitron shaker at 200 rpm (Informs HT, Swiss). Antibiotics were purchased from Sigma Aldrich (Merck, USA) except for zeocin (InvivoGen, USA).

### Plasmids and primers

Plasmids used in this study (Supplementary Table S2) are derivates of pMBA, a replicon containing a p15A origin of replication and a zeocin resistance gene. pMBA provides the genetic environment of cassettes in first position of a class 1 integron. It contains the 331 bp upstream the cassette, including the P_C_ (we chose the strong version of the P_C_ (34)) and P_int_ promoters, the *attI1* site (including ORF11), and the beginning of the integrase gene. A truncated integrase was used to avoid the deleterious effects of integrase expression and recombination.

To build all pMBA derivatives, we amplified the 5′UTRs from a collection of AgR gene cassettes available in the lab. When this was not possible, fragments containing 5′UTR-*gfp* fusions were synthesized *in vitro* (IDT, USA). Constructions were performed using Gibson Assembly (35). All constructions reproduced the sequence that would result from integrase mediated insertion of the cassette.

### Inducibility of *aacA5*

In an effort to recreate *Jia et al*.’s experiments, we studied the inducibility of *aacA5* using disc diffusion tests and induction in broth.

Disc diffusion tests: Overnight cultures of pMBA, pMBA_5′-_*_aacA5_* and pBGT were adjusted to a 0.5 in the McFarland scale using saline solution. These solutions were further diluted 1:200 and seeded on MH agar plates by inundation (∼3 ml) for 3 min. After discarding the remainder of the inoculum and letting the plates dry, discs containing kanamycin (30 μg), sisomycin (30 μg) or arabinose (2 μg) were placed on top of the agar. Kanamycin disks were obtained from Oxoid while sisomicin and arabinose discs were prepared in house using commercial 6-mm sterile discs (Oxoid, UK) and powder reagents. Plates were incubated overnight at 37°C. Pictures were taken in a GelDoc apparatus using epilight for growth and UV transillumination for GFP fluorescence (BioRad, USA). Fluorescence pictures were taken using constant exposition values across all plates to avoid misinterpretations derived from exposition differences. Fluorescence profiles across the diameter of the plate were obtained using ImageJ plot profile tool (https://imagej.nih.gov/ij/).

Induction in broth: three independent colonies of pMBA and pMBA_5′-_*_aacA5_* were inoculated in LB and incubated at 37°C overnight. Cultures were then diluted 1:50 in fresh LB containing a range of inhibitory and subinhibitory concentrations of kanamycin (0.125–16 μg/ml) and 2 × 200 μl of each were placed in 96-well plates and incubated at 37°C without shaking. Growth and GFP expression were followed along 24 h using a Biotek Synergy HTX plate reader and BioStack plate feeder (Agilent, USA). Growth (OD_600_) and fluorescence intensity at 488 nm were measured every 15 min with prior shaking at 567 cpm for 10 s. Growth and fluorescence curves are the result of the mean of the two technical and three biological replicates (six points) of each strain. In an interpretation of the experiments performed by Jia *et al.* we also took a sample of these cultures after 1 h of incubation, diluted 1:20 in filtered saline solution and measured fluorescence using a Cytoflex-S flow cytometer (Beckman Coulter, US).

### Minimal inhibitory concentration (MIC) determination

The MIC of the strain containing pMBA was determined against kanamycin (1 μg/ml), sisomycin (0.125 μg/ml), amikacin (1 μg/ml), gentamicin (0.25 μg/ml), neomycin (8 μg/ml), trimethoprim (0.125 μg/ml), tetracycline (0.25 μg/ml) and ciprofloxacin (0.016 μg/ml). These MICs are assumed to be extrapolable to all pMBA derivatives since none of them contain additional resistance genes. MIC determination was performed following CLSI guidelines. Briefly, 10^5^ UFCs were inoculated in 200 μl of fresh MH with doubling dilutions of antibiotics and incubated overnight in 96-well plates at 37°C in static conditions. The MIC is the mode of three biological replicates for each antibiotic.

### Database exploration

To describe all different AR cassettes, the content of the IntegrAll database (www.integrall.bio.ua.pt) was examined in November 2018. Applying a cutoff of 95% in cassette nucleotidic sequence, we recovered 177 different AR cassettes of which 64 were AgR genes.

To determine the frequency of appearance of each cassette gene family in the first position of class 1 integrons, the IntegrAll database was examined again in June 2021, retrieving 1927 distinct class 1 mobile integrons.

### Expression of AgR genes in resting conditions

Three independent colonies of each strain containing a pMBA derivative were inoculated in LB broth and incubated at 37°C overnight. A strain containing the pBGT plasmid in which GFP expression is under the control of a P_BAD_ promoter, was included as a landmark of expression. Three biological replicates of pBGT were incubated in the presence of glucose, arabinose and in the absence of supplements. All overnight cultures were diluted 1:400 in filtered saline solution and fluorescence intensity was measured by flow cytometry using a CytoFLEX-S cytometer (Beckman Coulter, USA). The measurement of each biological replicate is the result of the mean of the fluorescence intensity of 20 000 events per sample. Data processing was performed with Cytexpert software.

### Induction ratios of all AgR genes in the presence of antibiotics

At least three independent colonies of each strain containing a pMBA derivative were inoculated in LB broth and incubated at 37°C overnight. Overnight cultures were then diluted 1:50 in fresh LB with and without antibiotics at 1/2 the MIC of the strain carrying pMBA. Cultures were then incubated 1 h at 37°C with shaking (200 rpm), diluted 1:20 in filtered saline solution, and analyzed by flow cytometry. Experiments using trimethoprim were performed in LB and MH to decouple the activity from the presence of the molecule. To deliver the induction experiments with high concentrations of kanamycin, *armA* was cloned in a pBAD and transformed in a subset of strains. Induction with kanamycin at 128 μg/ml was performed as explained above in the presence of 0.1% l-arabinose throughout all experiments to ensure the expression of *armA*. Additionally, growth and GFP expression were monitored along 18 h using a Biotek Synergy HTX plate reader. Growth (OD_600_) and fluorescence intensity at 488 nm were measured every 15 min with prior shaking at 567 cpm for 10 s. Growth and fluorescence curves were obtained from three biological replicates of each strain. The induction ratio along the curve was calculated at each time point as the ratio (fluorescence/OD_600_) with kanamycin over (fluorescence/OD_600_) without kanamycin.

### Western blot

The strains showing the highest and lowest induction ratios in kanamycin were selected for relative GFP quantitation through western blot. Strains were incubated in LB at 37°C overnight. Cultures were then diluted 1:50 in fresh media with and without kanamycin at 0.5 μg/ml (1/2 the MIC of pMBA), and incubated at 37°C with shaking until an OD_600_ of 0.2 was reached. One ml of cells was pelleted, resuspended in 50 μl of Laemli buffer (BioRad) with 20% β-mercaptoethanol (BioRad), and boiled for 10 min. Cell lysates were then spun down and 5 μl of the supernatants were run on 4–12% gradient polyacrylamide precast gels (Invitrogen, USA). Following electrophoresis, proteins were transferred to nitrocellulose membrane using iBlot2 system (Invitrogen). Membranes were blocked with 2% BSA and 0.2% Tween-20 TBS for 1 h (ThermoFisher Scientific, Spain). Primary antibody incubation was performed during 2 h at room temperature with shaking. Primary antibodies used were mouse a-GFP 1:50 000 (Invitrogen) and mouse a-DnaK 1:6000 (Enzo Life Sciences, USA), in fresh 2% BSA TBS-T. Membranes were washed with 0.2% TBS–Tween (three times, 10 minutes each with shaking) and incubated at room temperature for 1 h with HRP-labelled goat anti-mouse IgG (Invitrogen) (secondary antibody) diluted 1:10 000 in fresh 2% BSA TBS-T. Images were obtained using SuperSignal West Pico Plus Chemiluminescent Substrate (Thermo Scientific) and ChemiDoc XRS+ imaging system (Bio-Rad, US). Data were analyzed using ImageLab software to quantify band intensities.

### Growth curves

Three independent colonies were inoculated in LB and incubated overnight at 37°C with shaking. Cultures were then diluted 1:50 in fresh LB or MH media and 200 μl were inoculated in 96-well plates. Plates were incubated for 20 h and OD_600_ was measured in 20 min-intervals using an incubated Synergy HTX plate reader. Plates were shaken at 280 cycles per minute before acquisition.

## RESULTS

### Cassettes in Class 1 integrons encode unrelated functions

Cassette mobility is a strong argument against the biological meaning of an Ag-sensing riboswitch. This mobility goes way beyond the reshuffling of positions within the same platform and deserves a deeper look into it. A wealth of data in the field put forward that mobile integrons recruit their cassettes from sedentary chromosomal integrons in environmental bacteria. It is remarkable that cassettes can be exchanged between different integrons. While integrases have co-evolved with their cognate *attI* sites and recognize specifically their sequence (and not that of other *attI* sites), recognition patterns of *attC* sites remain universal to all integrons (36,37). This is possible because *attC* sites in cassettes are not recognized for their sequence, but for their conserved hairpin-like structure (38–40). Cassettes are therefore streamlined to be exaptable and exchanged between evolutionarily distant integron platforms (30,41,42). It is of note that AR genes represent only a very small fraction of the myriad cassettes available in SCIs and whose functions remain mostly cryptic (37,43,44). The enrichment of AR cassettes in mobile integrons is likely related to the selective pressure with antibiotics exerted in the clinics.

**Figure 2.**
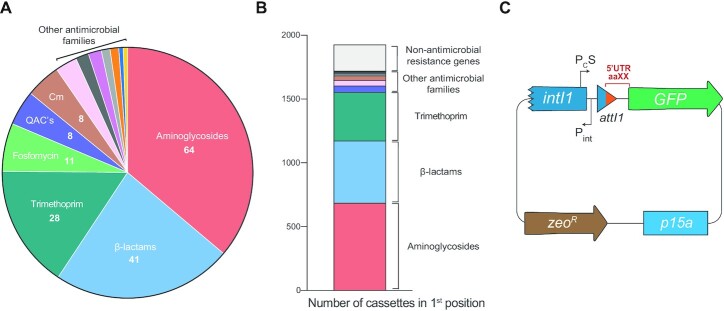
Mobile integrons contain cassettes encoding varied functions. (**A**) Distribution of resistance cassettes found in mobile integrons in the Integrall database (QACs: quaternary ammonium compounds; Cm: chloramphenicol). (**B**) Distribution of functions encoded in cassettes in first position of class 1 integrons in Integrall. (**C**) Diagram of pMBA vector used to analyse cassette expression by fusing 5′UTRs to a GFP encoding gene. Not to scale.

Given cassette mobility, the variety of functions found in cassettes also argues against the biological rationale behind an Ag-sensing riboswitch that is composed mostly (47/75 bp) of the *attI1* site of a class 1 integron, since the expression of unrelated genes would be contingent on the presence of aminoglycosides. To give an idea of this variety and unveil a potential bias in cassette order, we have analysed the functions encoded in the cassettes of MIs from the IntegrAll database (45) (www.integrall.bio.ua.pt). In line with previous reports (46), we have found 177 different resistance cassettes against most antibiotic families -not only aminoglycosides (Figure 2A). AgR genes are in first position of the array (next to the *attI*) in only 35% of class 1 integrons of the database, which correlates well with their relative abundance (36%: 64/177) and suggests there is no particular bias on the function encoded in first position. Indeed, genes conferring resistance against other antibiotics are also found frequently in first position (ß-lactams: 25%; trimethoprim: 20%; other families: 8.7%) together with cassettes not related to resistance or even cassettes of unknown function (10%) (Figure 2B). Hence, we could not find a bias towards the first position for cassettes that could potentially use the *attI1* site to form a riboswitch.

### Expression of *aacA5* is not Induced by aminoglycosides in its native genetic context

**Figure 3.**
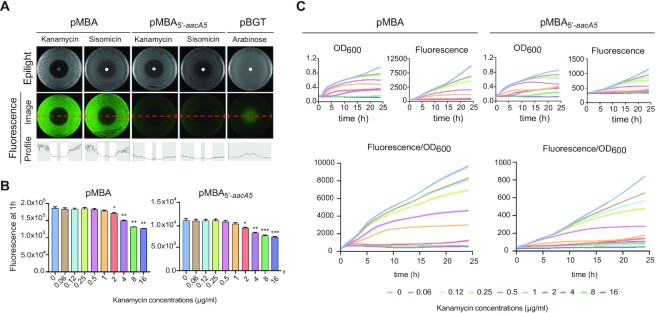
The expression of *aacA5* is not induced by aminoglycosides in its native genetic context. (**A**) Diffusion antibiograms of pMBA_5′-_*_aacA5_* in the presence of sisomycin and kanamycin. The profile does not show a fluorescent halo around the disc, similarly to the pMBA negative control, and in contrast to the pBGT positive control confronted to an arabinose disc. (**B**) GFP fluorescence in broth experiments after 1 hour of induction with kanamycin. Expression of *aacA5* decreases with increasing concentrations of antibiotic for both pMBA_5′-__*aacA5*_ and pMBA. Statistically significant differences compared to the no-antibiotic condition are marked with * (*P* < 0.05), **(*P* < 0.01) and ***(*P* < 0.001). (**C**) Growth (OD_600_), fluorescence (arbitrary units) and cassette expression (measured as fluorescence/OD_600_) along 24 h. A similar decrease in *aacA5* expression with increasing concentrations of kanamycin is observed along the whole growth curve.

The genetic context of any cassette is inherently variable, depending on the composition of the array and its position within it. The only sequence that all cassettes within a given integron platform share is the *attI* site where they are first inserted. This region is key to address the expression of cassettes because it contains the P_C_ promoter that can vary in strength (47), and small ORFs that can enhance translation, like ORF-11 in the *attI1* site. Hence, to provide the ideal genetic background to test the riboswitch hypothesis, we have designed pMBA, a p15A-based replicon bearing a class 1 integron platform and a *gfp* gene mimicking a cassette in first position, located immediately downstream the *attI* site (Figure 2C). pMBA contains a truncated version of the *intI1* gene to avoid the well-known deleterious effect of integrase activity, but conserves the P_C_S (the strong version of the P_C_) and P_int_ promoters and the *attI1* site. This is the native genetic context of cassettes in class 1 integrons and is a more biologically relevant genetic environment than the cloning vector used in Jia *et al.*’s work in which expression is controlled by an inducible P_lac_, preceded by the *lacI* repressor gene. The 5′ UTR of the *gfp* in pMBA (the nucleotides between the *attI* site and the start codon of the *gfp* gene) is not related to any integron cassette and contains a strong ribosome binding site (RBS). To test the behavior of *aacA5* in the presence of aminoglycosides, we exchanged this region by the 5′ UTR of *aacA5*, obtaining pMBA_5′-_*_aacA5_*. We subjected this construction to induction with kanamycin and sisomicin in diffusion antibiograms, in an effort to recreate Jia *et al.*’s experiments in which they used a beta-galactosidase reporter instead (Figure 3A). As controls, we exposed a strain containing pMBA to both antibiotics, and a strain containing the pBGT plasmid—in which the *gfp* gene is under the control of a P_BAD_ promoter (48)—to an arabinose-containing disc to induce its expression. Results with pBGT validated the assay and provided a plot with a clear inducibility profile. pMBA_5′-_*_aacA5_* did not show increased levels of fluorescence at the border of the inhibition halos, as would have been expected from a riboswitch-mediated induction of expression, and instead had a profile similar to that of the pMBA control.

We have also recreated Jia *et al.*’s induction experiments in broth, by growing strains carrying pMBA and pMBA_5′-_*_aacA5_* in doubling concentrations of kanamycin ranging from subinhibitory to inhibitory and measuring fluorescence after 1 h through flow cytometry (Figure 3B). We did not observe an increase in fluorescence at any concentration. Instead, fluorescence started decreasing as antibiotic concentrations approached the minimum inhibitory concentration (MIC) of our strain (1 μg/ml). It is of note that the bacterial inoculum used in these experiments was higher than the one used to determine the MIC (see Materials and Methods), which explains the visible growth at concentrations above the MIC (a well-known phenomenon named inoculum effect (49)). To test whether a longer time-period was necessary to observe an induction, we analysed growth and expression levels in all concentrations of kanamycin for 24 h (Figure 3C). Our data revealed an inhibitory effect in growth at all concentrations that was overlooked in Jia *et al.*’s experiments and could be relevant since it seems to affect bacterial physiology. We took it into account by normalizing fluorescence to optical density. Data showed that the expression of *gfp* was not induced in the presence of kanamycin at any concentration tested. Instead, fluorescence (both raw, and normalized by the OD) was lower in the presence of the antibiotic than in its absence in agreement with previous results. We observed a similar behavior for the empty pMBA control, suggesting that the decrease in fluorescence/OD_600_ is a consequence of the general inhibition of protein synthesis caused by kanamycin rather than a specific effect on the 5′ UTR of *aacA5*. Altogether, our data do not support a riboswitch-controlled model of expression for the cassette *aacA5*.

### Building an exhaustive collection of AgR cassettes

**Figure 4.**
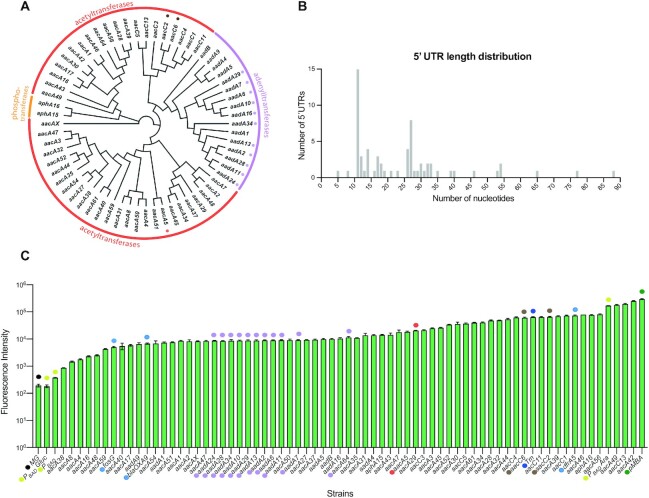
Aminoglycoside resistance cassettes are not repressed in the absence of antibiotics. (**A**) Tree showing the phylogenetic relationship of the 64 AgR proteins found in integron cassettes. The enzymatic activity is marked with colored lines. Proteins encoded in cassettes with identical 5′ UTRs are marked with mauve and brown dots, and pMBA_5′-_*_aacA5_* is marked with a red dot. (**B**) Size distribution of 5′ UTRs within AgR cassettes. (**C**) Expression of AgR cassettes in resting conditions measured as the fluorescence of 5′UTR-*gfp* fusions. Expression levels of the P_BAD_ promoter are depicted as landmarks of expression (yellow dots). Controls include the MG1655 strain without the *gfp* gene (MG: black dot), pMBA (green dot), cassettes conferring resistance to unrelated antibiotics (light blue dots) and the thermometer riboswitch (dark blue dot). As in panel A, constructions with identical 5′ UTRs are marked with mauve and brown dots, and pMBA_5′-_*_aacA5_* with a red dot. See also Supplementary Table S1 and Supplementary Figure S1.

The expression of *aacA5* is, in our hands, not induced by kanamycin or sisomycin. Yet it is possible that other AgR genes in integrons possess such control mechanisms, as suggested in other works from the same group. We hence sought to analyze the inducibility of all known AgR cassettes. To do this, we explored the Integrall database and retrieved AgR cassettes in mobile integrons using a 95% identity cutoff in nucleotide sequence. We found 64 cassettes encoding aminoglycoside inactivating enzymes, including members of the acetyltransferase (46), adenylyltransferase (16) and phosphotransferase (2) protein families (Figure 4A). We synthesized all cassettes and, as we did for pMBA_5′-_*_aacA5_*, we inserted their 5′ UTRs between the *attI* site and the *gfp* start codon in pMBA (sequences are available in Supplementary Table S1). These UTRs were defined as the sequence starting at the TT of the 5′-GTT-3′ triplet (where the recombination crossover takes place in integrons (Figure 1A)) to the start codon of the gene. We used gene and *attC* site annotations from IntegrAll, but in the case of cassettes with potentially dubious annotations, alternative (alt) UTRs were also tested (see below). Among our UTRs we could find most of the sequences from Murchie's works, such as the one of *aacA5* from (14), which is identical to aac-1 in (15); and those of aac-2, 3 and 4 from (15) -here *aacA27*, *aacA3* and *aacA4* alt, respectively. Finally, the UTR of *aadB* is identical to the UTRs of 5 out of 6 *aad* sequences in (16), but in that work the authors included, as part of the putative riboswitch, bases from the coding regions of the resistance genes. The 5′UTRs selected here varied considerably in size from 3 to 86 bp (Figure 4B). Eleven out of 13 *aadA* cassettes in our collection had the same 5′ UTR, but were cloned and treated independently as biological replicates (mauve dots in Figure 4A and onwards). This was also the case for the 5′UTRs of *aacC2* and *aacC6* (brown dots in Figure 4A). This provides a control of technical and biological variability in our assays. Once this library of 5′ UTRs from AgR cassettes was established and verified, we included as additional controls: (i) the pMBA vector, (ii) a plasmid-less MG1655 strain that does not contain a *gfp* gene (MG); (iii) three constructions with the 5′ UTRs of integron cassettes that confer resistance to unrelated antibiotics -such as trimethoprim (*dfrA5*), fosfomycin (*fosG*), and beta-lactams (*bla*_OXA-9_); and (iv) a thermometer riboswitch that responds to temperature, not antibiotics (50).

### Aminoglycoside resistance cassettes are not repressed in the absence of antibiotics

To characterize the expression of AgR cassettes in resting conditions, we measured the fluorescence of all 5′ UTR-*gfp* fusions in the absence of antibiotics (Figure 4C). Fluorescence levels varied 170-fold between the least (pMBA_5′-__*aacA38*_) and the most expressed cassette (pMBA_5′-_*_aacA42_*), and the rest of cassettes were evenly distributed throughout this scale. To put these levels of expression in context, we have included as a landmark of expression a P_BAD_ promoter in repressed, unrepressed and induced conditions. This promoter is known to have a broad dynamic range with good levels of induction and repression. Cassettes with the highest expression levels were similar to the induced P_BAD_. Instead, the least expressed cassette was 10-fold higher than the repressed P_BAD_ or the no-GFP control (MG1655 strain). Cassettes conferring resistance against other antibiotics did not group together in a qualitatively distinct expression range, but instead were found among the least and the most expressed cassettes. Hence, the differences in expression levels among cassettes do not support a common repressing mechanism. Instead, expression levels correlated well (*R*^2^ = 0.69) with the presence of a Shine-Dalgarno sequence in the vicinity of the start codon (Supplementary Figure S1). The net expression levels of most—if not all—cassettes are incompatible with a biologically relevant repressed status, as one would expect for an uninduced riboswitch.

### Aminoglycosides do not induce the expression of aminoglycoside resistance cassettes

Aminoglycosides are a large family of antibiotics that can be classified in three sub-classes attending to their chemical structure: 4,5- and 4,6-disubstituted deoxystreptamines (-DD), and the non-deoxystreptamines. Most clinically relevant molecules like kanamycin, sisomicin, gentamicin or amikacin are 4,6-DDs, while neomycins and paramomycin are 4,5-DDs, and streptomycin is a non-DD. Induction of *aacA5* was shown to occur in the presence of 4,6- but not 4,5-DDs. Hence, despite questioning the inducing effect of these molecules in this work, for the sake of simplicity we will use ‘inducing’ Ags to refer to 4,6-DDs and ‘non-inducing’ for 4,5-DDs.

We sought to better verify the inducibility of all AgR cassettes by aminoglycosides by broadening the molecules tested to include the inducers gentamicin and amikacin. All antibiotics were used at $\frac{1}{2}$ of the MIC to provide conditions in which concentrations are maximal before strongly inhibiting bacterial growth. As a control, we included the non-inducing aminoglycoside neomycin. Correct growth was verified for all conditions (Supplementary Figure S2).

**Figure 5.**
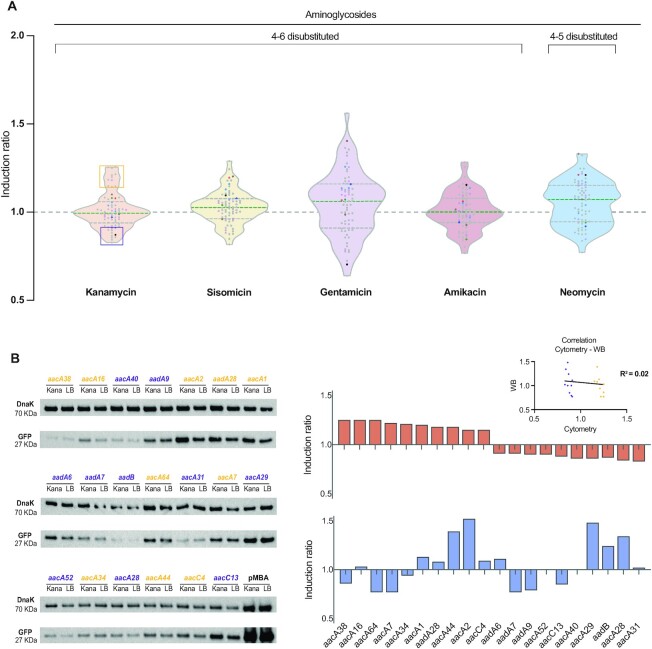
Aminoglycosides do not induce the expression of aminoglycoside resistance cassettes. (**A**) Violin plots showing the induction ratios of all cassettes in the presence of four inducing aminoglycosides, and the non-inducing neomycin. Color code of the dots as in Figure 4. Median and quartiles are represented by dashed lines. Linear regression analysis did not find significant differences (*P* > 0.3) in fluorescence readings in the absence versus the presence of Ags. A linear mixed model revealed statistically significant differences for gentamicin and amikacin, but they were mild, and in opposite direction (see text and Supplementary Figure S4). This suggests that aminoglycosides do not induce the expression of AgR cassettes. Each data point represents the mean of at least three measurements. (**B**) Induction ratio measured through Western blot of the 10 cassettes with highest and lowest IRs in kanamycin measured by flow cytometry (orange and violet boxes in panel A). GFP abundance was normalized to DnaK. Cytometry and Western blot results do not correlate (inset: linear regression *R*^2^= 0.02), suggesting that the distribution of IRs in cytometry does not reflect a biologically relevant induction (i.e. these values are not the consequence of a specific interaction between the antibiotic and the 5′ UTRs of this subset of genes). See also Supplementary Figures S2–S4.

We calculated the induction ratio (IR) of all cassettes as the ratio between the fluorescence in the presence over the absence of the antibiotic, so that non-inducible genes should have an IR around 1, while inducible genes should have an IR > 1. As an example, the IR of the P_BAD_ promoter in Figure 4C is 170. IRs of all cassettes showed distributions with a median close to 1 in the presence of inducer aminoglycosides (0.99 for kanamycin, 1.05 for sisomicin, 1 for amikacin and 1.07 for gentamicin) (Figure 5A). The non-inducer neomycin showed the highest median of induction (1.077), although very similar to that of gentamicin. Maximal and minimal IR values observed were around 1.3 and 0.8 respectively for all Ags except for gentamicin that showed a higher dispersion (1.56–0.63). We also tested alternative UTRs for cassettes with potentially dubious annotations, and showed a similar non-inducible profile (Supplementary Figure S3). The small increase observed even for maximal IR values argues against a biologically relevant induction for any gene. Instead, the distribution of IR values suggests that variations are likely due to noise, whether biological or technical. To address this, we examined more closely the 11 constructions with identical UTRs (from *aad* alleles) that were treated as independent biological replicates. Their IR values showed differences that comprised a large part of the variation in the whole dataset (Figure 5A, mauve dots). Taking kanamycin as an example, the IR values of 76% (49/64) AgR cassettes fell between the highest and lowest IR of these identical UTRs. If we include the controls that are not related to AgR, this figure reached 81% for kanamycin and sisomycin, 72% for gentamicin, 87% for amikacin and 84% for neomycin. This strongly suggests that the variability found among genes is non-specific, and that it most likely reflects biological or technical noise, and/or the pleiotropic effect of the antibiotic. We performed a statistical analysis of our data comparing fluorescence levels through linear regression. We did not find significant differences in the presence of an aminoglycoside compared with the baseline readings in the absence of antibiotic (*P* > 0.3). We then applied a linear mixed model accounting for genes and experimental batches as random effects, and observed significant changes in the fluorescence readings in the presence of gentamicin and amikacin, but not kanamycin, sisomycin nor neomycin (Supplementary Figure S4, Supplementary Table S1). These effects were extremely mild, with a mean increase in expression of 5% for gentamicin, and a mean decrease of 7.5% for amikacin. Altogether, the lack of coherent effects among inducer molecules and of biological significance of the induction ratios, rules out the presence of a common regulatory mechanism capable of sensing aminoglycosides and inducing gene expression.

Despite the lack of a clear induction across the whole set of AgR genes, one could still argue that not all cassettes have riboswitches but that those with the highest IR values are precisely the subset of them that do. To address this, we have quantitated GFP protein in the presence and the absence of kanamycin using Western blot (WB) (Figure 5B and Supplementary Figure S4). This experiment uses the quantitation of another protein (DnaK) as an internal control for normalization, which is key to establish if the variation in GFP levels is specific to its 5′ UTR, or if it is consequence of a general trend in the cell's proteome. To determine this, we have analyzed ten cassettes showing the highest IR values with kanamycin, and have compared them to the ten cassettes with the lowest IRs (orange and violet boxes in the kanamycin violin plot in Figure 5A). Cytometry and WB data clearly show a lack of correlation (*R*^2^ = 0.02) (inset in Figure 5B) suggesting that genes with high IRs in cytometry do not represent a distinct subset of cassettes with a specific induction mechanism.

### Higher concentrations of aminoglycosides neither induce resistance genes

**Figure 6.**
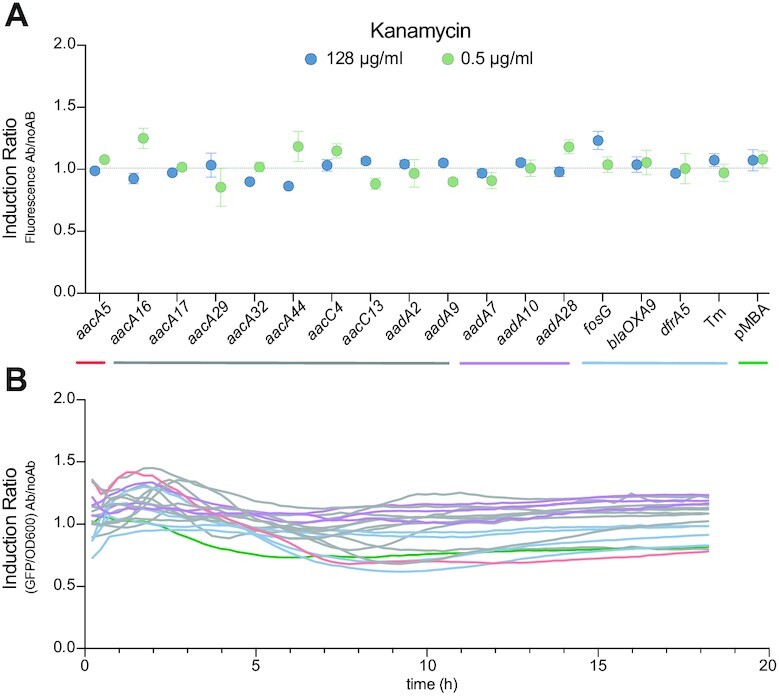
High concentrations of aminoglycosides do not induce AgR cassettes. (**A**) Induction ratio of a subset of AgR cassettes in the presence of 128 μg/ml of kanamycin (blue dots). As a reference, the IR values at 0.5 μg/ml of kanamycin from Figure 5 are also represented (green dots). (**B**) Induction ratio (calculated as the ratio of fluorescence/OD_600_ with kanamycin over fluorescence/OD_600_ without kanamycin) of these strains along their growth curve (18 h). Color code is as in (A). Green: pMBA; red: pMBA_5′-_*_aacA5_*; blue: control strains; mauve: strains with identical 5′-UTRs; gray: rest of strains.

The concentrations of antibiotics used for *in vivo* experiments in the first report of the putative riboswitch are close or above the minimal inhibitory concentration of a susceptible *E. coli* K-12 derivative such as their (and our) strain. Indeed, the highest induction level they observe for kanamycin is at 2.4 μg/ml (5 μM), in a strain with a MIC around 1 μg/ml. A similar situation is found in follow up works where, concentrations are also above the MIC (15,16). Accordingly, it is mentioned that all these experiments are delivered titrating the antibiotics, a rather uncommon practice in the field of resistance. We hence decided to address whether riboswitches could be acting at concentrations above the MIC, despite not conforming to a biologically sound rationale. To do so, we introduced the AgR gene *armA* in a subset of our strains. ArmA is a 16S rRNA methylase that provides high levels of resistance against 4–6-DDs, but does not modify the antibiotic. Crucially, *armA* is not encoded in an integron cassette (51). We repeated induction experiments of cassettes in the presence of 256-fold higher concentrations of kanamycin and confirmed that they remain uninduced (Figure 6).

### Induction is higher in the presence of unrelated antibiotics

**Figure 7.**
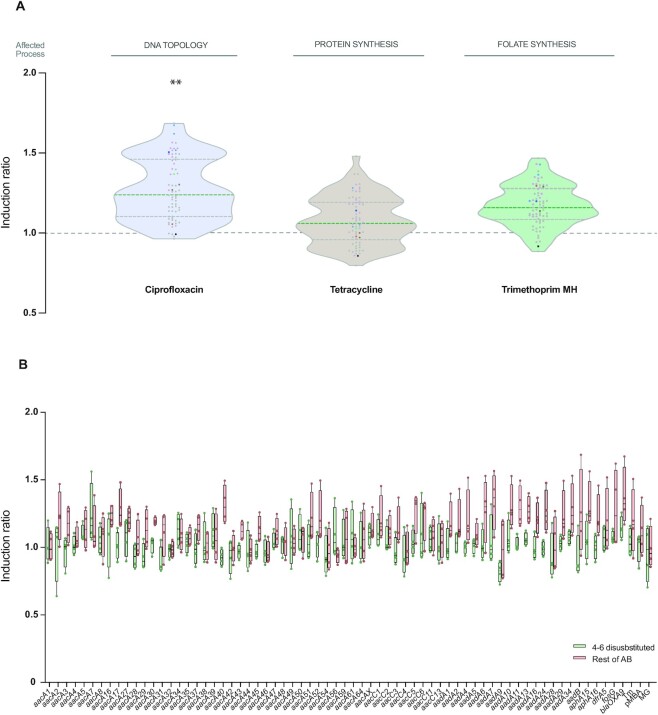
Induction is higher in the presence of unrelated antibiotics. (**A**) Violin plots showing the induction ratio of all genes in the presence of non-aminoglycoside antibiotics. Color code as in Figure 4. Median and quartiles are represented by dashed lines. Although low, median IR values are consistently higher for these unrelated antibiotics than for aminoglycosides. Ciprofloxacin increases the expression of constructions in a statistically significant manner (*P* = 0.006). Each data point represents the mean of at least three measurements. (**B**) Representation, for each AgR gene, of the IR in the presence of inducing aminoglycosides (green) and non-inducing antibiotics–neomycin and non-aminoglycosides (red). Inducer aminoglycosides do not have a consistent nor biologically relevant effect in the expression of any cassette. Non-inducer antibiotics show a stronger induction effect than inducer aminoglycosides in 53 out of 64 genes and these differences are significative for 10 of them. See also Supplementary Figure S2 and S4.

Our experiments at $\frac{1}{2}$ the MIC show that aminoglycosides can have a very mild and non-specific influence in the measures of IRs. To provide a broader context to our data we have tested the effect of unrelated antibiotics in the induction of all 5′ UTRs (Figure 7A). We chose molecules with different modes of action, such as tetracycline -a protein synthesis inhibitor, like aminoglycosides, but with a distinct mode of action; ciprofloxacin -a topoisomerase inhibitor- and trimethoprim -that inhibits the biosynthesis of folate. With these antibiotics, the medians of IR were all higher than with aminoglycosides (tetracycline: 1.08; ciprofloxacin: 1.26; trimethoprim: 1.19), and estimated differences in our linear mixed model due to the antibiotic were larger than those associated with the addition of aminoglycosides (Supplementary Figure S4). No construction showed an IR > 2 in any antibiotic, and >93% of the IR values of AgR cassettes fell within those of control strains, pointing to a non-specific effect of these molecules on the expression of AgR cassettes.

To address if there is any cassette with a mild but consistent inducible behaviour across our experiments, we analysed independently the IRs of each UTR in the presence of inducing aminoglycosides (4,6-DDs) and non-inducing antibiotics (4,5-DDs and unrelated antibiotics) (Figure 7B). Our data clearly shows that 4–6-DDs do not induce the expression of AgR cassettes to biologically relevant levels in any case. The stronger effects of other antibiotics are also evident in this analysis since 83% (53/64) of the genes had higher median IRs in the presence of non-inducing molecules compared to 4–6-DDs, and statistically significant differences were observed in 10 of them.

### Increased expression is due to pleiotropic effects of antibiotics

**Figure 8.**
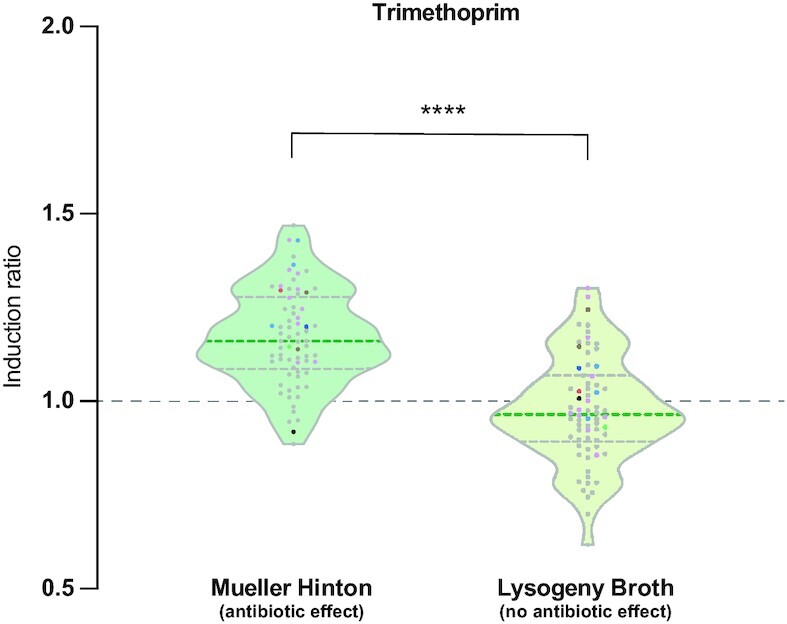
Increased expression is due to pleiotropic effects of antibiotics. Induction ratio of all cassettes in the presence of trimethoprim in LB compared to MH (data from Figure 7). In LB, where trimethoprim does not have antibiotic effect, median IRs are lower than in MH (0.96 instead of 1.16) and this difference is statistically significant (*P* > 0.0001). This confirms that small increases in IRs can be the consequence of pleiotropic effects of unrelated antibiotics. See also Supplementary Figure S2 and S4.

As mentioned above, Roth and Breaker suggested the mild induction observed by Murchie and collaborators could be due to pleiotropic effects of antibiotics (33). Our data strongly support this claim. Conveniently, we can test this experimentally because the presence of trimethoprim can be decoupled from its antibiotic effect, by measuring IRs in Lysogeny Broth (LB), instead of Müeller Hinton (MH). In contrast to MH, LB contains traces of thymine that render the folate route redundant so that trimethoprim is not lethal in LB. We analyzed the induction of all cassettes in the presence of trimethoprim in LB and compared them to our previous results in MH (Figure 8). The observed mean of IR values decreased from 1.17 to 0.98 in the absence of antibiotic and this effect was statistically significant using both linear regression and a random linear mixed model (*P* < 0.0001). This confirms that pleiotropic effects of antibiotics can alter the expression of genes 1.5-fold range in a non-specific manner. This is approximately the range observed by Jia *et al.*

## DISCUSSION

A deep understanding on antibiotic resistance mechanisms is necessary to predict the emergence and spread of resistant bacteria. This is especially the case for critically important antibiotics—like aminoglycosides—and for regulatory processes that can help alleviate the cost of resistance in the absence of selection. In this context, the presence of Ag-sensing riboswitches is a relevant finding that needed yet to be confirmed experimentally by independent research groups.

Apart from the counterintuitive involvement of the *attI1* site in the putative riboswitch structure, and other incisive arguments provided by Roth and Breaker on the narrow induction range, there are other aspects of Jia *et al.*’s initial report that justified to our eyes the need for an independent assessment. First, the *aacA5* cassette provides a key part of the riboswitch (the SD/anti-SD switch) in its 25 bp-long 5′ UTR. Our analysis reveals that 37 of the 64 AgR genes in integrons contain UTR’s shorter than 25 bp (Figure 4B). This makes the sequestering of the SD in resting conditions impossible for many UTRs, and underscores that the possibility of a conserved mechanism among AgR cassettes is intrinsically flawed. Second, the concentrations of antibiotics used for *in vivo* experiments are close or above the minimal inhibitory concentration of the strain. As mentioned previously, these experiments are delivered titrating the antibiotics, a rather uncommon practice in the field of resistance. Also, these concentrations are in sharp contrasts with reports of aminoglycosides possibly acting as signaling molecules and having an impact in many aspects of cell physiology at concentrations as low as 1–2% of the MIC, and where titration of the antibiotic is unnecessary (52–54). Using a subset of resistant strains exposed to extremely high concentrations of antibiotics, we could not observe changes in GFP expression. Hence, the lack of induction observed in our experiments is not due to low antibiotic concentrations. Third, as a negative control of induction by aminoglycosides, the authors use the leader sequence of the chloramphenicol resistance gene *cat86*. This sequence is known to be regulated by a ribosome stalling mechanism (5) and adds an additional level of repression to the system. It therefore seems inadequate to be used as a single control of non-inducibility by aminoglycosides. Fourth, in-line probing and Surface Plasmon Resonance (SPR) results need to be interpreted considering the antibiotic concentrations used. For instance, in crosslinking experiments, concentrations of sisomycin (100 μM = 44.7 μg/ml) are ∼350-fold higher than the MIC of a susceptible *E. coli* as the one used in our experiments (MIC = 0.125 μg/ml). Similarly, probing experiments in the presence of 100 μM kanamycin are ∼50-fold above the MIC. It is of note that binding to RNA (to the ribosomal 16S RNA) is the mode of action of aminoglycosides, and that spurious interactions can occur and are probably favored at the extreme concentrations used by Jia *et al.* Indeed, it has been shown that these molecules can bind negatively charged pockets in RNA structures involved in processes where this binding is biologically irrelevant (55,56). As pointed by Roth and Breaker in their letter, the fact that the most prominent site of structure modulation by aminoglycosides is the SD2, which is outside the *attI1* and shows little conservation, is consistent with spurious Ag-RNA interactions, rather than the specific binding to a conserved, putative riboswitch. It is also noteworthy that, based on SPR results, Jia *et al.* argue that inducer molecules show higher affinity for the putative riboswitch than non-inducer molecules, but this claim is not supported by their results. They use as examples the dissociation constants (*K*_D_) of the inducers tobramycin, kanamycin and sisomycin (2.19, 2.78 and 6.8 μM, respectively) and compare them to those of non-inducers ribostamycin, neamine and paromomycin (*K*_D_ of 589, 47 and 12 μM respectively). Yet in their Figure 4, amikacin, netilmicin and gentamicin—which are all inducers—have a *K*_D_ of 35.2, 32.6 and 12.7 μM, respectively. These values are in a range similar to those of neamine and paromomycin, and leads to the rather counterintuitive conclusion that inducers and non-inducers can have similar affinities for the putative riboswitch. In any case, it seems altogether clear that both *in vivo* and *in vitro* results lack physiological relevance since the riboswitch should sense and react to the antibiotic at concentrations well below the MIC in order to induce the expression of the resistance gene in a timely manner. Fifth and last, we have shown here that genes conferring resistance against other antibiotics—and even unknown functions—are found frequently in first position of class 1 integrons (Figure 2B). This contrasts with the almost universal presence of aminoglycoside resistance genes in first position reported by the authors in several works (14–16). The difference stems from the fact that they report the results of BLAST searches using as query the sequence of an aminoglycoside resistance gene fused to the *attI1*, hence biasing the list of hits towards such genes.

Altogether, these concerns led us to investigate the presence of riboswitches in AgR cassettes. Here we provide data on the inducibility of all AgR genes encoded in integron cassettes in the presence of a variety of antibiotics. A relevant aspect of our work is that we study cassette expression in its genetic environment, conserving the 331 bp found upstream any newly acquired cassettes in a class 1 integron. This sequence contains all structures potentially able to influence cassette expression, such as the P_C_ and P_int_ promoters and the whole *attI1* site including the small ORF-11 that enhances the expression of genes with poor translation initiation regions (57). We argue that this is the appropriate setting to measure cassette expression and a more biologically relevant one than the general-purpose cloning vector used in the reports on Ag-riboswitches, in which expression is repressed by LacI. In this genetic context, we were unable to replicate the induction results for *aacA5* using the methodologies in (14). We then broadened our approach to all 5′ UTRs of AgR cassettes -including several genes used in the follow up articles- and used single-cell resolution (cytometry) to measure inducibility. We combined this with relative quantification through Western blots, showing that variation in GFP intensity is not specific of the UTR in question. WB can lack sensitivity to quantitate protein abundance in single samples, yet the clear lack of correlation with cytometry results across groups of isolates strongly supports the lack of specific induction by aminoglycosides. This, together with the fact that induction ratios were higher using non-aminoglycoside antibiotics, pointed, as suggested by Roth and Breaker, to pleiotropic effects of antibiotics as responsible for variation in expression. We finally proved this by measuring the induction ratios of our constructs with trimethoprim in conditions were its presence and its antibiotic effect could be uncoupled. Altogether, our results rule out the existence -in integron AgR cassettes- of a regulatory mechanism that induces gene expression in the presence of aminoglycosides. It therefore challenges the validity of the Ag-sensing riboswitch.

## Supplementary Material

gkac662_Supplemental_Files

## References

[1] Andersson D.I. , Hughes D. Antibiotic resistance and its cost: is it possible to reverse resistance. Nat. Rev. Microbiol. 2010; 8:260–271. 20208551 10.1038/nrmicro2319

[2] Foucault M.L. , Depardieu F., Courvalin P., Grillot-Courvalin C. Inducible expression eliminates the fitness cost of vancomycin resistance in enterococci. Proc. Nat. Acad. Sci. U.S.A. 2010; 107:16964–16969. 10.1073/pnas.1006855107 PMC2947908 20833818

[3] Foucault M.L. , Courvalin P., Grillot-Courvalin C. Fitness cost of vana-type vancomycin resistance in methicillin-resistant *staphyloco**ccus**aureus*. Antimicrob. Agents Chemother. 2009; 53:2354–2359. 19332680 10.1128/AAC.01702-08 PMC2687198

[4] Morosini M.I. , Ayala J.A., Baquero F., Martinez J.L., Blazquez J. Biological cost of AmpC production for *salmonella**enterica* serotype typhimurium. Antimicrob. Agents Chemother. 2000; 44:3137–3143. 11036037 10.1128/aac.44.11.3137-3143.2000 PMC101617

[5] Alexieva Z. , Duvall E.J., Ambulos N.P., Kim U.J., Lovett P.S. Chloramphenicol induction of *cat-86* requires ribosome stalling at a specific site in the leader. Proc. Nat. Acad. Sci. U.S.A. 1988; 85:3057–3061. 10.1073/pnas.85.9.3057 PMC280142 3129723

[6] Weisblum B. , Siddhikol C., Lai C.J., Demohn V. Erythromycin-inducible resistance in *staphylococcus**aureus*: requirements for induction. J. Bacteriol. 1971; 106:835–847. 4397638 10.1128/jb.106.3.835-847.1971 PMC248701

[7] Reilman E. , Mars R.A.T., van Dijl J.M., Denham E.L. The multidrug ABC transporter BmrC/BmrD of *bacillus**subtilis* is regulated via a ribosome-mediated transcriptional attenuation mechanism. Nucleic Acids Res. 2014; 42:11393–11407. 25217586 10.1093/nar/gku832 PMC4191407

[8] Dar D. , Shamir M., Mellin J.R., Koutero M., Stern-Ginossar N., Cossart P., Sorek R. Term-seq reveals abundant ribo-regulation of antibiotics resistance in bacteria. Science. 2016; 352:aad9822-1. 27120414 10.1126/science.aad9822 PMC5756622

[9] Evers S. , Courvalin P. Regulation of vanb-type vancomycin resistance gene expression by the VanSB-VanRB two-component regulatory system in *enterococcus**faecalis* V583. J. Bacteriol. 1996; 178:1302–1309. 8631706 10.1128/jb.178.5.1302-1309.1996 PMC177803

[10] Normark S. ß-lactamase induction in Gram-negative bacteria is intimately linked to peptidoglycan recycling. Microb Drug Resist. 1995; 1:111–114. 9158742 10.1089/mdr.1995.1.111

[11] Da Re S. , Garnier F., Guérin E., Campoy S., Denis F., Ploy M.C. The SOS response promotes *qnrB* quinolone-resistance determinant expression. EMBO Rep. 2009; 10:929–933. 19556999 10.1038/embor.2009.99 PMC2726673

[12] Levin B.R. Minimizing potential resistance: a population dynamics view. Clin. Infect. Dis. 2001; 33:161–169. 10.1086/321843 11524714

[13] World Health Organization Critically Important Antimicrobials for Human Medicine, 6th revision. 2019; Geneva.

[14] Jia X. , Zhang J., Sun W., He W., Jiang H., Chen D., Murchie A.I.H. Riboswitch control of aminoglycoside antibiotic resistance. Cell. 2013; 152:68–81. 23332747 10.1016/j.cell.2012.12.019

[15] Wang S. , He W., Sun W., Zhang J., Chang Y., Chen D., Murchie A.I.H. Integron-derived aminoglycoside-sensing riboswitches control aminoglycoside acetyltransferase resistance gene expression. Antimicrob. Agents Chemother. 2019; 63:e00236-19. 30936094 10.1128/AAC.00236-19 PMC6535548

[16] Zhang J. , Liu G., Zhang X., Chang Y., Wang S., He W., Sun W., Chen D., Murchie A.I.H. Aminoglycoside riboswitch control of the expression of integron associated aminoglycoside resistance adenyltransferases. Virulence. 2020; 11:1432–1442. 33103573 10.1080/21505594.2020.1836910 PMC7588185

[17] Partridge S.R. , Recchia G.D., Scaramuzzi C., Collis C.M., Stokes H.W., Hall R.M. Definition of the *attl1* site of class 1 integrons. Microbiology. 2000; 146:2855–2864. 11065364 10.1099/00221287-146-11-2855

[18] Recchia G.D. , Hall R.M. Gene cassettes: a new class of mobile element. Microbiology. 1995; 141:3015–3027. 8574395 10.1099/13500872-141-12-3015

[19] Stokes H.W. , Hall R.M. A novel family of potentially mobile DNA elements encoding site-specific gene-integration functions: integrons. Mol. Microbiol. 1989; 3:1669–1683. 2560119 10.1111/j.1365-2958.1989.tb00153.x

[20] Escudero J.A. , Loot C., Nivina A., Mazel D. The integron: adaptation on demand. Microbiol. Spectrum. 2015; 3:MDNA3–A0019–2014. 10.1128/microbiolspec.MDNA3-0019-2014 26104695

[21] Mazel D. Integrons: agents of bacterial evolution. Nat. Rev. Microbiol. 2006; 4:608–620. 16845431 10.1038/nrmicro1462

[22] Recchia G.D. , Stokes H.W., Hall R.M. Characterisation of specific and secondary recombination sites recognised by the integron DNA integrase. Nucleic Acids Res. 1994; 22:2071–2078. 8029014 10.1093/nar/22.11.2071 PMC308123

[23] Francia M.V. , Zabala J.C., De La Cruz F., García Lobo J.M. The IntI1 integron integrase preferentially binds single-stranded DNA of the *attC* site. J. Bacteriol. 1999; 181:6844–6849. 10542191 10.1128/jb.181.21.6844-6849.1999 PMC94154

[24] Demarre G. , Frumerie C., Gopaul D.N., Mazel D. Identification of key structural determinants of the IntI1 integron integrase that influence *attC* × *attI1* recombination efficiency. Nucleic Acids Res. 2007; 35:6475–6489. 17884913 10.1093/nar/gkm709 PMC2095811

[25] Collis C.M. , Hall R.M. Expression of antibiotic resistance genes in the integrated cassettes of integrons. Antimicrob. Agents Chemother. 1995; 39:155–162. 7695299 10.1128/aac.39.1.155 PMC162502

[26] Souque C. , Escudero J.A., MacLean R.C. Integron activity accelerates the evolution of antibiotic resistance. Elife. 2021; 10:e62474. 33634790 10.7554/eLife.62474 PMC8024014

[27] Baharoglu Z. , Bikard D., Mazel D. Conjugative DNA transfer induces the bacterial SOS response and promotes antibiotic resistance development through integron activation. PLoS Genet. 2010; 6:e1001165. 20975940 10.1371/journal.pgen.1001165 PMC2958807

[28] Barraud O. , Ploy M.C. Diversity of class 1 integron gene cassette rear-rangements selected under antibiotic pressure. J. Bacteriol. 2015; 197:2171–2178. 25897031 10.1128/JB.02455-14 PMC4455274

[29] Guerin É. , Cambray G., Sanchez-Alberola N., Campoy S., Erill I., Re S.Da, Gonzalez-Zorn B., Barbé J., Ploy M.C., Mazel D. The SOS response controls integron recombination. Science. 2009; 324:1034. 19460999 10.1126/science.1172914

[30] Rowe-Magnus D.A. , Guerout A.M., Ploncard P., Dychinco B., Davies J., Mazel D. The evolutionary history of chromosomal super-integrons provides an ancestry for multiresistant integrons. Proc. Nat. Acad. Sci. U.S.A. 2001; 98:652–657. 10.1073/pnas.98.2.652 PMC14643 11209061

[31] Cury J. , Jové T., Touchon M., Néron B., Rocha E.P. Identification and analysis of integrons and cassette arrays in bacterial genomes. Nucleic Acids Res. 2016; 44:4539–4550. 27130947 10.1093/nar/gkw319 PMC4889954

[32] Ghaly T.M. , Chow L., Asher A.J., Waldron L.S., Gillings M.R. Evolution of class 1 integrons: mobilization and dispersal via food-borne bacteria. PLoS One. 2017; 12:e0179169. 28586403 10.1371/journal.pone.0179169 PMC5460862

[33] Roth A. , Breaker R.R. Integron *attI1* sites, not riboswitches, associate with antibiotic resistance genes. Cell. 2013; 153:1417. 23791167 10.1016/j.cell.2013.05.043 PMC4626886

[34] Vinué L. , Jové T., Torres C., Ploy M.C. Diversity of class 1 integron gene cassette pc promoter variants in clinical *escherichia**coli* strains and description of a new P2 promoter variant. Int. J. Antimicrob. Agents. 2011; 38:526–529. 21917427 10.1016/j.ijantimicag.2011.07.007

[35] Gibson D.G. , Young L., Chuang R.Y., Venter J.C., Hutchison C.A., Smith H.O. Enzymatic assembly of DNA molecules up to several hundred kilobases. Nat. Methods. 2009; 6:343–345. 19363495 10.1038/nmeth.1318

[36] Biskri L. , Bouvier M., Guérout A.M., Boisnard S., Mazel D. Comparative study of class 1 integron and *vibrio**cholerae* superintegron integrase activities. J. Bacteriol. 2005; 187:1740–1750. 15716446 10.1128/JB.187.5.1740-1750.2005 PMC1063995

[37] Rowe-Magnus D.A. , Guerout A.M., Mazel D. Bacterial resistance evolution by recruitment of super-integron gene cassettes. Mol. Microbiol. 2002; 43:1657–1669. 11952913 10.1046/j.1365-2958.2002.02861.x

[38] Stokes H.W. , O’Gorman D.B., Recchia G.D., Parsekhian M., Hall R.M. Structure and function of 59-base element recombination sites associated with mobile gene cassettes. Mol. Microbiol. 1997; 26:731–745. 9427403 10.1046/j.1365-2958.1997.6091980.x

[39] Bouvier M. , Ducos-Galand M., Loot C., Bikard D., Mazel D. Structural features of single-stranded integron cassette *attC* sites and their role in strand selection. PLoS Genet. 2009; 5:e1000632. 19730680 10.1371/journal.pgen.1000632 PMC2727003

[40] MacDonald D. , Demarre G., Bouvier M., Mazel D., Gopaul D.N. Structural basis for broad DNA-specificity in integron recombination. Nature. 2006; 440:1157–1162. 16641988 10.1038/nature04643

[41] Rowe-Magnus D.A. , Guerout A.M., Biskri L., Bouige P., Mazel D. Comparative analysis of superintegrons: engineering extensive genetic diversity in the vibrionaceae. Genome Res. 2003; 13:428–442. 12618374 10.1101/gr.617103 PMC430272

[42] Ghaly T.M. , Tetu S.G., Gillings M.R. Predicting the taxonomic and environmental sources of integron gene cassettes using structural and sequence homology of *attC* sites. Commun.Biol. 2021; 4:946. 34373573 10.1038/s42003-021-02489-0 PMC8352920

[43] Boucher Y. , Labbate M., Koenig J.E., Stokes H.W. Integrons: mobilizable platforms that promote genetic diversity in bacteria. Trends Microbiol. 2007; 15:301–309. 17566739 10.1016/j.tim.2007.05.004

[44] Rapa R.A. , Labbate M. The function of integron-associated gene cassettes in *vibrio* species: the tip of the iceberg. Front. Microbiol. 2013; 4:385. 24367362 10.3389/fmicb.2013.00385 PMC3856429

[45] Moura A. , Soares M., Pereira C., Leitão N., Henriques I., Correia A. INTEGRALL: a database and search engine for integrons, integrases and gene cassettes. Bioinformatics. 2009; 25:1096–1098. 19228805 10.1093/bioinformatics/btp105

[46] Partridge S.R. , Tsafnat G., Coiera E., Iredell J.R. Gene cassettes and cassette arrays in mobile resistance integrons. FEMS Microbiol. Rev. 2009; 33:757–784. 19416365 10.1111/j.1574-6976.2009.00175.x

[47] Jové T. , Da Re S., Denis F., Mazel D., Ploy M.C. Inverse correlation between promoter strength and excision activity in class 1 integrons. PLoS Genet. 2010; 6:1000793. 10.1371/journal.pgen.1000793 PMC2791841 20066027

[48] San Millan A. , Escudero J.A., Gifford D.R., Mazel D., MacLean R.C. Multicopy plasmids potentiate the evolution of antibiotic resistance in bacteria. Nat. Ecol. Evol. 2016; 1:0010. 10.1038/s41559-016-0010 28812563

[49] Tan C. , Phillip Smith R., Srimani J.K., Riccione K.A., Prasada S., Kuehn M., You L. The inoculum effect and band-pass bacterial response to periodic antibiotic treatment. Mol. Syst. Biol. 2012; 8:617. 23047527 10.1038/msb.2012.49 PMC3472685

[50] Weber G.G. , Kortmann J., Narberhaus F., Klose K.E. RNA thermometer controls temperature-dependent virulence factor expression in *vibrio**cholerae*. Proc. Natl. Acad. Sci. U.S.A. 2014; 111:14241–14246. 25228776 10.1073/pnas.1411570111 PMC4191814

[51] González-Zorn B. , Catalan A., Domínguez L., Teshager T., Porrero C., Moreno M.A. Genetic basis for dissemination of *armA*. J. Antimicrob. Chemother. 2005; 56:583–585. 16027145 10.1093/jac/dki246

[52] Baharoglu Z. , Mazel D. *Vibrio* *cholerae* triggers SOS and mutagenesis in response to a wide range of antibiotics: a route towards multiresistance. Antimicrob. Agents Chemother. 2011; 55:2438–2441. 21300836 10.1128/AAC.01549-10 PMC3088271

[53] Baharoglu Z. , Babosan A., Mazel D. Identification of genes involved in low aminoglycoside-induced SOS response in *Vibrio**cholerae*: a role for transcription stalling and mfd helicase. Nucleic Acids Res. 2014; 42:2366–2379. 24319148 10.1093/nar/gkt1259 PMC3936754

[54] Carvalho A. , Krin E., Korlowski C., Mazel D., Baharoglu Z. Interplay between sublethal aminoglycosides and quorum sensing: consequences on survival in *V. cholerae*. Cells. 2021; 10:3227. 34831448 10.3390/cells10113227 PMC8621022

[55] Hermann T. , Westhof E. Docking of cationic antibiotics to negatively charged pockets in RNA folds. J. Med. Chem. 1999; 42:1250–1261. 10197968 10.1021/jm981108g

[56] Hermann T. , Westhof E. Aminoglycoside binding to the hammerhead ribozyme: a general model for the interaction of cationic antibiotics with RNA. J. Mol. Biol. 1998; 276:903–912. 9566195 10.1006/jmbi.1997.1590

[57] Hanau-Berçot B. , Podglajen I., Casin I., Collatz E. An intrinsic control element for translational initiation in class 1 integrons. Mol. Microbiol. 2002; 44:119–130. 11967073 10.1046/j.1365-2958.2002.02843.x

