# Supplementary material for: The expression of aminoglycoside resistance genes in integron cassettes is not controlled by riboswitches
Source: Nucleic Acids Res. 2022 Aug 10;50(15):8566–79. doi: 10.1093/nar/gkac662 (PMC9410878; doi:10.1093/nar/gkac662)
Supplement: gkac662_Supplemental_Files [file gkac662_supplemental_files.zip › Figure S1_merged-2.pdf]

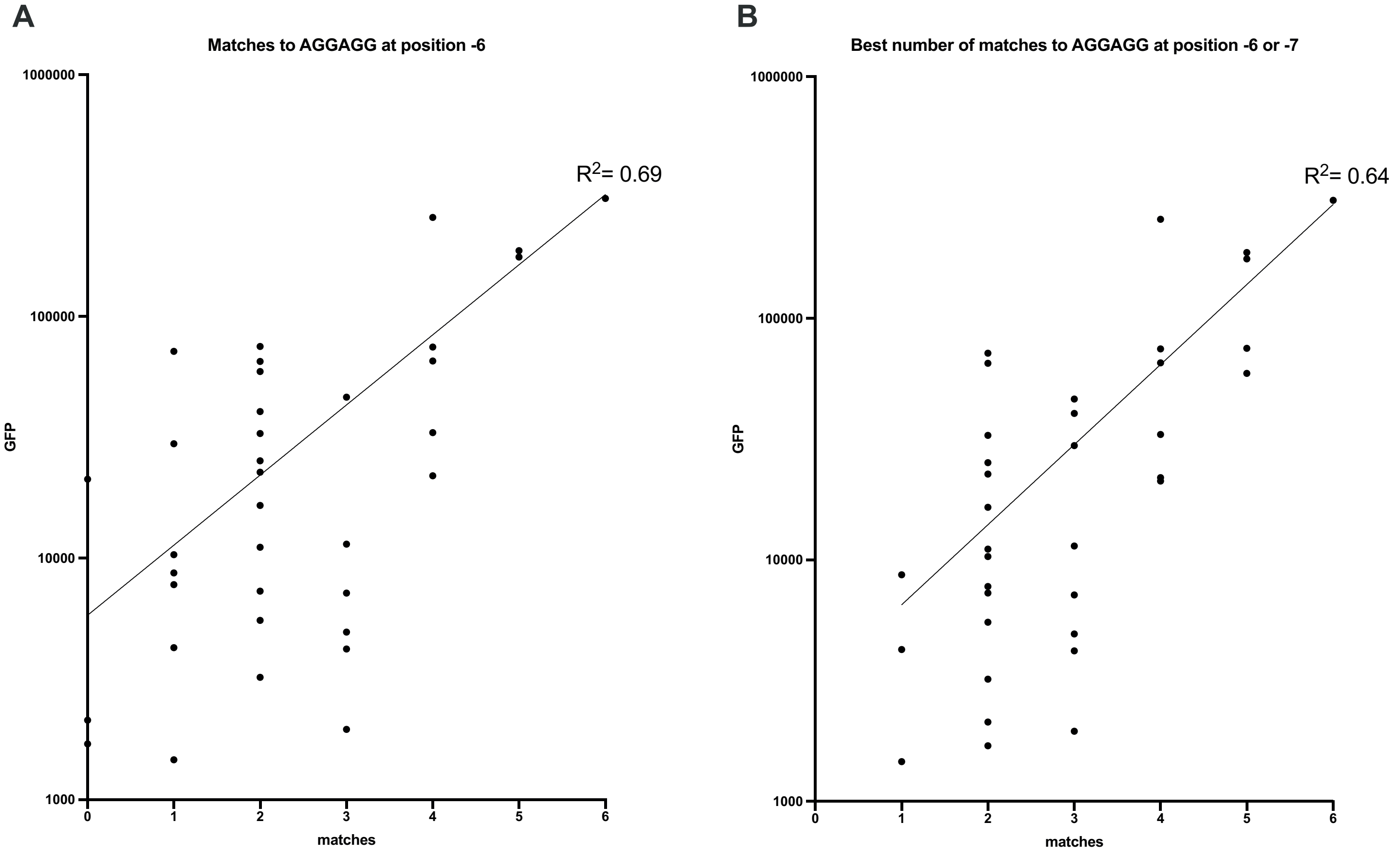

Figure S1. Cassette expression correlates with the presence of Shine Dalgarno-like sequences. Related to Figure 4

Correlation between GFP fluorescence and the number of matches to AGGAGG at a distance of (A) 6 bp from the start codon (positions -7 to -12); and (B) 6 or 7 bp from the start codon (positions -7 to -13). Curves were fitted using a non linear (semilog) regression. 5'-UTRs that are too short to encode an RBS at these positions were excluded from the analysis.

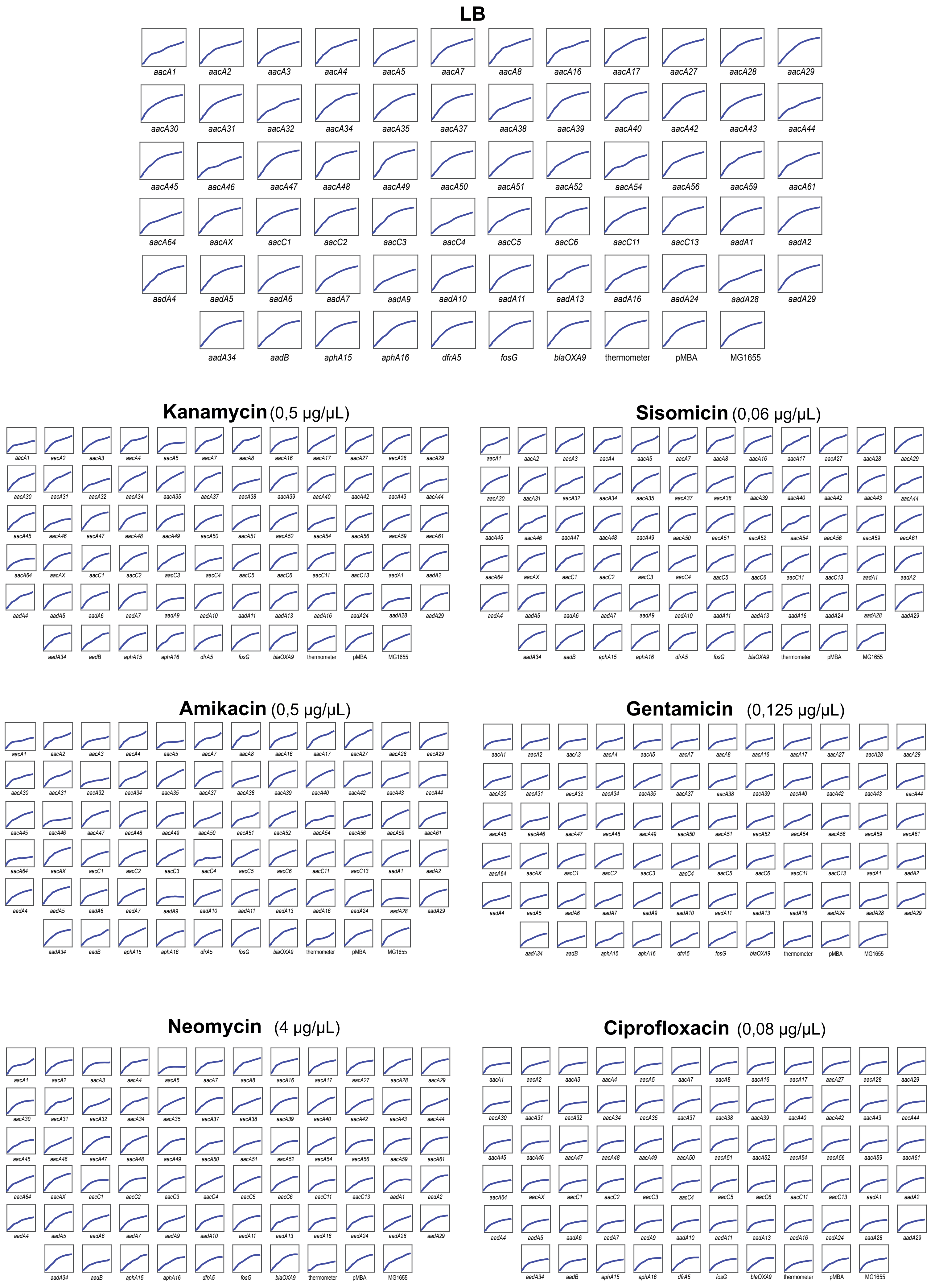
LB
aacA1 aacA2 aacA3 aacA4 aacA5 aacA7 aacA8 aacA16 aacA17 aacA27 aacA28 aacA29
aacA30 aacA31 aacA32 aacA34 aacA35 aacA37 aacA38 aacA39 aacA40 aacA42 aacA43 aacA44
aacA45 aacA46 aacA47 aacA48 aacA49 aacA50 aacA51 aacA52 aacA54 aacA56 aacA59 aacA61
aacA64 aacAX aacC1 aacC2 aacC3 aacC4 aacC5 aacC6 aacC11 aacC13 aadA1 aadA2
aadA4 aadA5 aadA6 aadA7 aadA9 aadA10 aadA11 aadA13 aadA16 aadA24 aadA28 aadA29
aadA34 aadB aphA15 aphA16 dfrA5 fosG blaOXA9 thermometer pMBA MG1655
Kanamycin (0,5 µg/µL)
aacA1 aacA2 aacA3 aacA4 aacA5 aacA7 aacA8 aacA16 aacA17 aacA27 aacA28 aacA29
aacA30 aacA31 aacA32 aacA34 aacA35 aacA37 aacA38 aacA39 aacA40 aacA42 aacA43 aacA44
aacA45 aacA46 aacA47 aacA48 aacA49 aacA50 aacA51 aacA52 aacA54 aacA56 aacA59 aacA61
aacA64 aacAX aacC1 aacC2 aacC3 aacC4 aacC5 aacC6 aacC11 aacC13 aadA1 aadA2
aadA4 aadA5 aadA6 aadA7 aadA9 aadA10 aadA11 aadA13 aadA16 aadA24 aadA28 aadA29
aadA34 aadB aphA15 aphA16 dfrA5 fosG blaOXA9 thermometer pMBA MG1655
Sisomicin (0,06 µg/µL)
aacA1 aacA2 aacA3 aacA4 aacA5 aacA7 aacA8 aacA16 aacA17 aacA27 aacA28 aacA29
aacA30 aacA31 aacA32 aacA34 aacA35 aacA37 aacA38 aacA39 aacA40 aacA42 aacA43 aacA44
aacA45 aacA46 aacA47 aacA48 aacA49 aacA50 aacA51 aacA52 aacA54 aacA56 aacA59 aacA61
aacA64 aacAX aacC1 aacC2 aacC3 aacC4 aacC5 aacC6 aacC11 aacC13 aadA1 aadA2
aadA4 aadA5 aadA6 aadA7 aadA9 aadA10 aadA11 aadA13 aadA16 aadA24 aadA28 aadA29
aadA34 aadB aphA15 aphA16 dfrA5 fosG blaOXA9 thermometer pMBA MG1655
Amikacin (0,5 µg/µL)
aacA1 aacA2 aacA3 aacA4 aacA5 aacA7 aacA8 aacA16 aacA17 aacA27 aacA28 aacA29
aacA30 aacA31 aacA32 aacA34 aacA35 aacA37 aacA38 aacA39 aacA40 aacA42 aacA43 aacA44
aacA45 aacA46 aacA47 aacA48 aacA49 aacA50 aacA51 aacA52 aacA54 aacA56 aacA59 aacA61
aacA64 aacAX aacC1 aacC2 aacC3 aacC4 aacC5 aacC6 aacC11 aacC13 aadA1 aadA2
aadA4 aadA5 aadA6 aadA7 aadA9 aadA10 aadA11 aadA13 aadA16 aadA24 aadA28 aadA29
aadA34 aadB aphA15 aphA16 dfrA5 fosG blaOXA9 thermometer pMBA MG1655
Gentamicin (0,125 µg/µL)
aacA1 aacA2 aacA3 aacA4 aacA5 aacA7 aacA8 aacA16 aacA17 aacA27 aacA28 aacA29
aacA30 aacA31 aacA32 aacA34 aacA35 aacA37 aacA38 aacA39 aacA40 aacA42 aacA43 aacA44
aacA45 aacA46 aacA47 aacA48 aacA49 aacA50 aacA51 aacA52 aacA54 aacA56 aacA59 aacA61
aacA64 aacAX aacC1 aacC2 aacC3 aacC4 aacC5 aacC6 aacC11 aacC13 aadA1 aadA2
aadA4 aadA5 aadA6 aadA7 aadA9 aadA10 aadA11 aadA13 aadA16 aadA24 aadA28 aadA29
aadA34 aadB aphA15 aphA16 dfrA5 fosG blaOXA9 thermometer pMBA MG1655
Neomycin (4 µg/µL)
aacA1 aacA2 aacA3 aacA4 aacA5 aacA7 aacA8 aacA16 aacA17 aacA27 aacA28 aacA29
aacA30 aacA31 aacA32 aacA34 aacA35 aacA37 aacA38 aacA39 aacA40 aacA42 aacA43 aacA44
aacA45 aacA46 aacA47 aacA48 aacA49 aacA50 aacA51 aacA52 aacA54 aacA56 aacA59 aacA61
aacA64 aacAX aacC1 aacC2 aacC3 aacC4 aacC5 aacC6 aacC11 aacC13 aadA1 aadA2
aadA4 aadA5 aadA6 aadA7 aadA9 aadA10 aadA11 aadA13 aadA16 aadA24 aadA28 aadA29
aadA34 aadB aphA15 aphA16 dfrA5 fosG blaOXA9 thermometer pMBA MG1655
Ciprofloxacin (0,08 µg/µL)
aacA1 aacA2 aacA3 aacA4 aacA5 aacA7 aacA8 aacA16 aacA17 aacA27 aacA28 aacA29
aacA30 aacA31 aacA32 aacA34 aacA35 aacA37 aacA38 aacA39 aacA40 aacA42 aacA43 aacA44
aacA45 aacA46 aacA47 aacA48 aacA49 aacA50 aacA51 aacA52 aacA54 aacA56 aacA59 aacA61
aacA64 aacAX aacC1 aacC2 aacC3 aacC4 aacC5 aacC6 aacC11 aacC13 aadA1 aadA2
aadA4 aadA5 aadA6 aadA7 aadA9 aadA10 aadA11 aadA13 aadA16 aadA24 aadA28 aadA29
aadA34 aadB aphA15 aphA16 dfrA5 fosG blaOXA9 thermometer pMBA MG1655

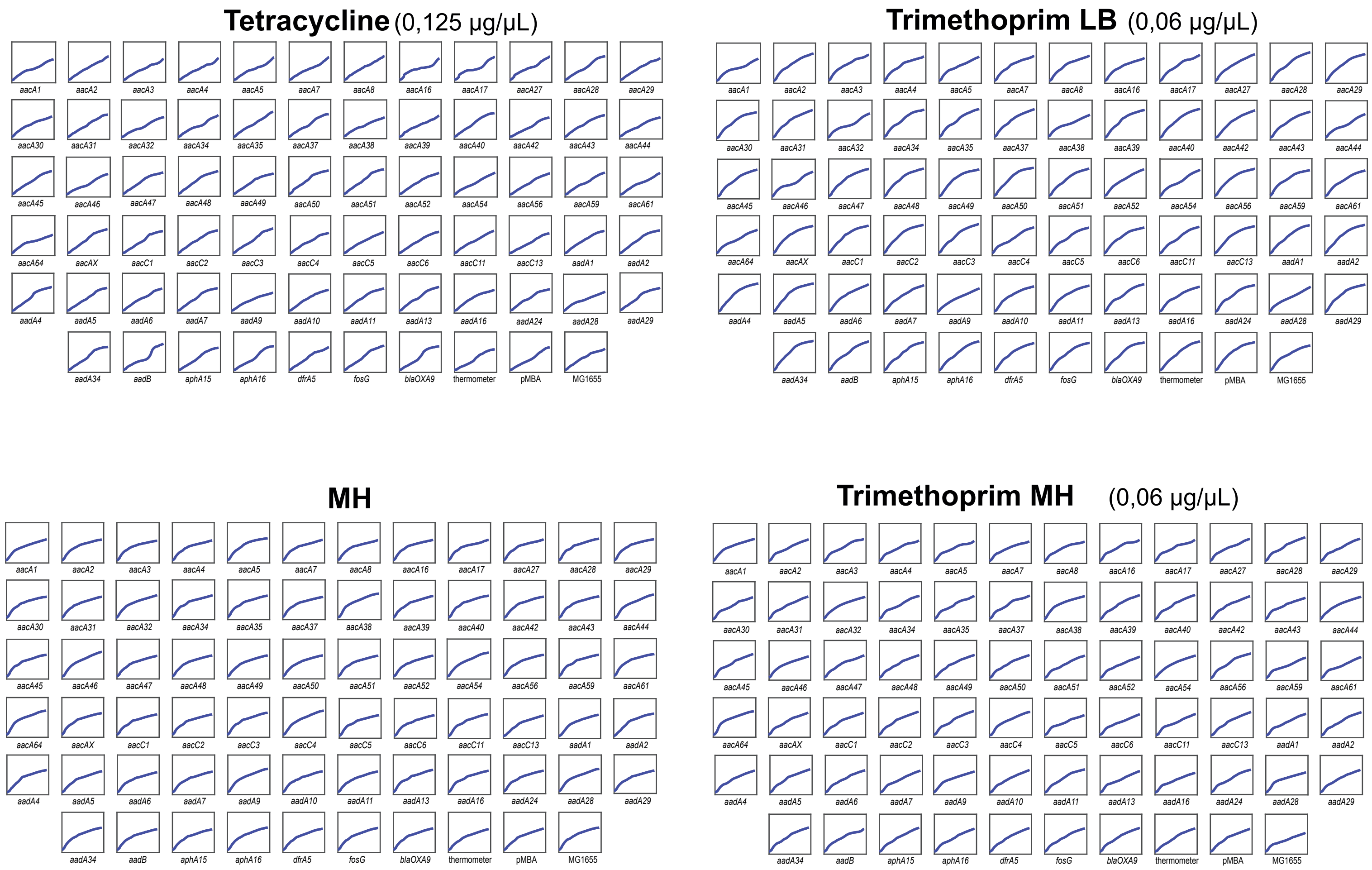

Figure S2. Antibiotic concentrations used do not affect growth, related to Figure 3. Growth curves of all constructions in the media used to deliver induction experiments. Curves are the mean of three replicates. The order of 5´UTRs is conserved among panels and can be seen in the large LB panel.

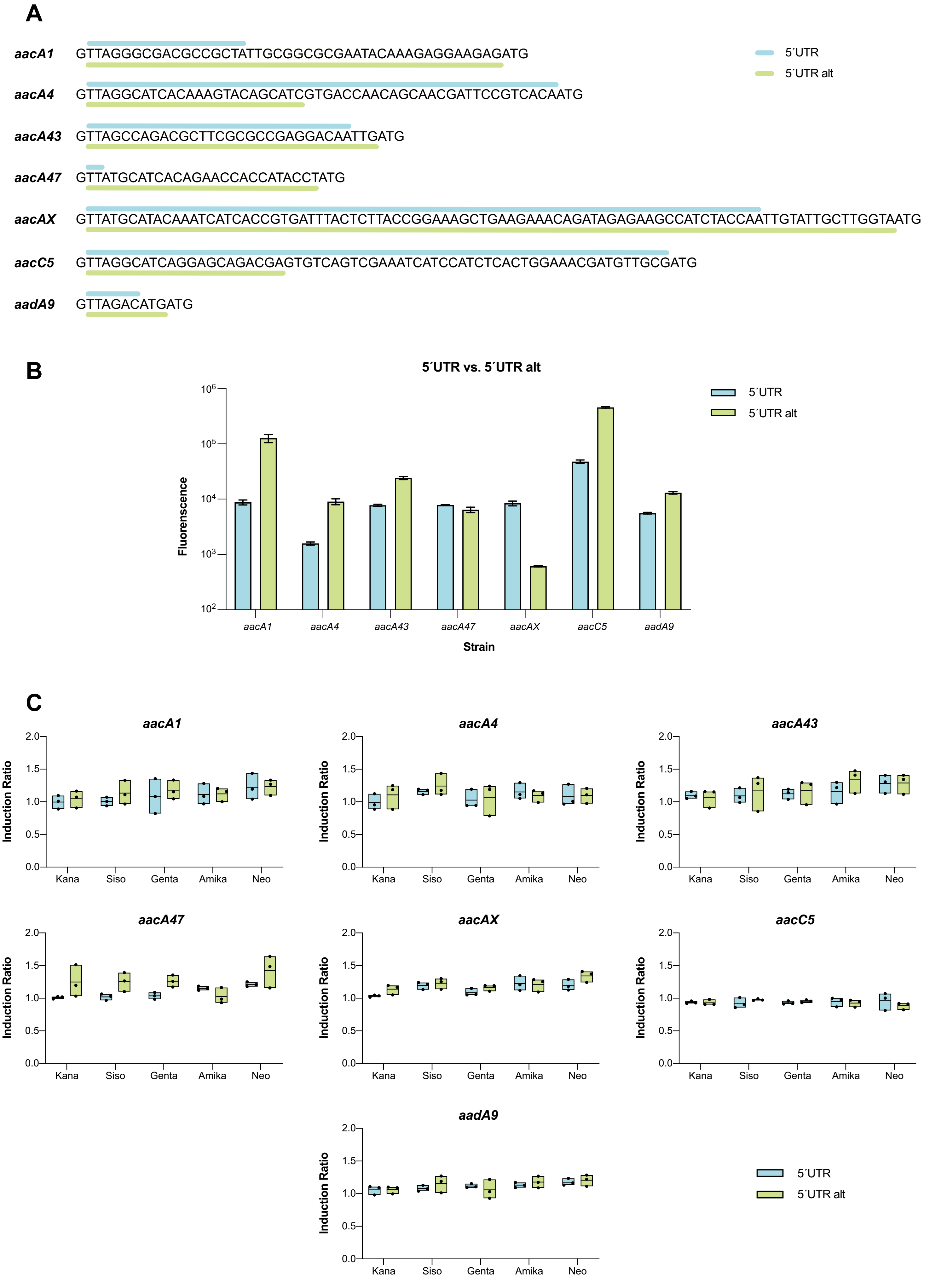

Figure S3. Alternative 5'UTRs are not induced by aminoglycosides, related to Figure 5. (A) Sequence of main and alternative 5'UTRs used in case of dubious annotations. (B) Fluorescence of main and alternative sequences fused to the GFP gene. (C) Induction ratios of main and alternative 5'UTRs for all aminoglycosides. Induction is not observed for any sequence.

A

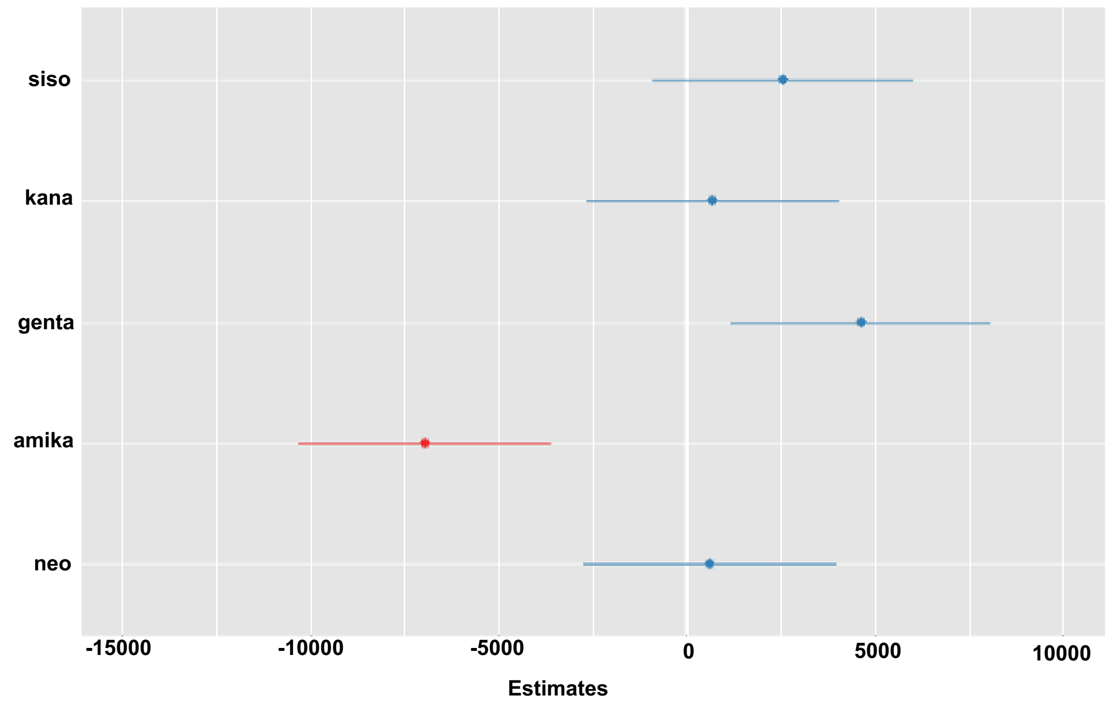

B

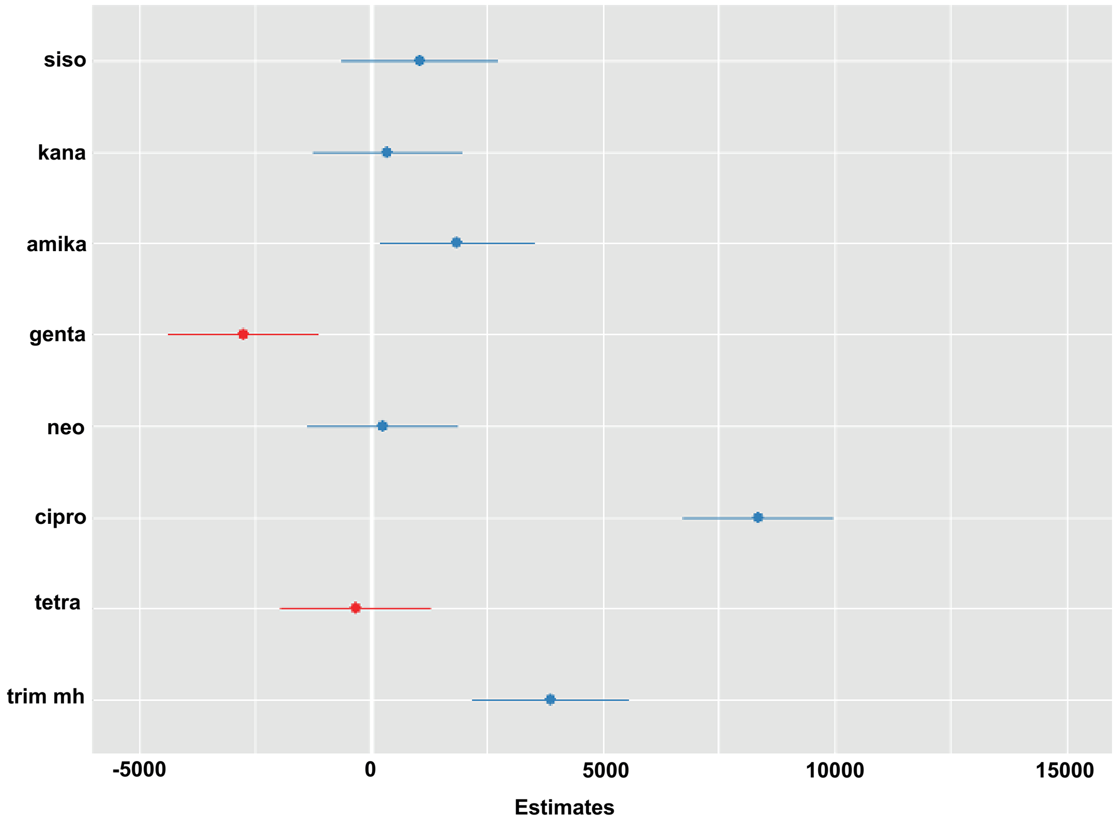

C

Antibiotic	Estimate	95% CI		p-value
		Lower bound	Upper bound	
Sisomicin[a]	1023.2	-359.1	2405.4	0.15
Kanamicin[a]	275.5	-1064.5	1614.2	0.67
Gentamicin[a]	1853.0	475.1	3230.9	0.009
Amikacin[a]	-2786.8	-4130.1	-1443.7	<0.001
Neomycin[a]	247.4	-1096.6	1591.3	0.72
Ciprofloxacin[b]	8348.0	6708.6	9993.0	<0.001
Tetracycline[b]	-331.72	-1972.3	1314.5	0.69
Trimethoprim[b]	3876.3	2196.8	5557.1	<0.001

[a] Coefficients from a model including only aminoglycosides
[b] Coefficients from a model including all antibiotics in the study

Figure S4, Results from statistical models, related to Figures 5 and 7. Change in fluorescence readings in the presence of (A) aminoglycosides and (B) all antibiotics, as determined by a linear mixed regression model considering gene, batch and sample as random effects. (C) Expected change in fluorescence readings after the addition of antibiotics as estimated by a linear mixed model including genes and batches as random effects.
