# Supplementary material for: The expression of aminoglycoside resistance genes in integron cassettes is not controlled by riboswitches
Source: Nucleic Acids Res. 2022 Aug 10;50(15):8566–79. doi: 10.1093/nar/gkac662 (PMC9410878; doi:10.1093/nar/gkac662)
Supplement: gkac662_Supplemental_Files [file gkac662_supplemental_files.zip › Table S1.docx]

**Table S1. Sequence of all integron cassette 5’UTRs used in this work, related to Figure 4.** The GTT crossover point is marked in blue, and the start codon of the GFP in green.

| **Resistance gene Cassette** | **5’ UTR Sequence** |
| --- | --- |
| *aacA1* | GTTAGGGCGACGCCGCTAATG |
| *aacA2* | GTTAGGCGTCATG |
| *aacA3* | GTTAGGCAGCACAGAGCGACCATTTCATG |
| *aacA4* | GTTAGGCATCACAAAGTACAGCATCGTGACCAACAGCAACGATTCCGTCACAATG |
| *aacA5* | GTTAGGCAGCACGGAGACACTTCAGCATG |
| *aacA7* | GTTAGGCACCAATG |
| *aacA8* | GTTAGGCAGCACAAACTCCGTCCTCATG |
| *aacA16* | GTTGGGCTGATTGATTTGTTTGTTCTAGCATTACCTATATG |
| *aacA17* | GTTGGGCTGATTGATTTGTTTGTTCTAGTATTACCTATATG |
| *aacA27* | GTTAGGCCCGCACGGAATCAACATCTCATG |
| *aacA28* | GTTAGCCGGACGCTGCGCGCGAAGAGGTTTTATG |
| *aacA29* | GTTAGACGGCTATG |
| *aacA30* | GTTAGGCTGGCGCGCTTCGCGCGGAAGACTTTATGGCTACTCGGAGACCTTAAATG |
| *aacA31* | GTTAGGCAGCACAAAGACCGTTCTCATG |
| *aacA32* | GTTAGGCAGCACAAAAGGACCGTCCCATG |
| *aacA34* | GTTAGAAGGCCCAGGCTATG |
| *aacA35* | GTTAGGCAGCACAGGGCCACCTTCTTATG |
| *aacA37* | GTTAACCGCGGCTATG |
| *aacA38* | GTTAGGCAGCACATAACCACCGTCACACCATG |
| *aacA39* | GTTAGCCGGACGCTTCGCGCAAAGAGGTATTATG |
| *aacA40* | GTTAGGCAGCACAGTCCAGACTCCGCATG |
| *aacA42* | GTTAGGCTGACGCGCTTCGCGCGGAAGACTTTATGGCTACTCGGAGACTTTGAATG |
| *aacA43* | GTTAGCCAGACGCTTCGCGCCGAGGACAAATG |
| *aacA44* | GTTAGGCAGCACAAGACCACCTGTTCATGCCCGCGAACGAAAACACCGTAACCCTACGTCTGATGACTGAGCACAATTGGTGATTAAATG |
| *aacA45* | GTTAAAAGGCTCAGCCAATG |
| *aacA46* | GTTAGGGCGACGCCGCATTCGCGGCGCGTGAAGAAAGAGGATCTTATG |
| *aacA47* | GTTATG |
| *aacA48* | GTTAGGTCCCACTAAACCTGCACCGAGCATG |
| *aacA49* | GTTAGCTTGACGCTTCGCGCAGAGGAGAGTTTCAATG |
| *aacA50* | GTTAGACAGCACAAAGACAATTCTCATG |
| *aacA51* | GTTAGGCCACAAGGAACCGTCCCAGTATG |
| *aacA52* | GTTAGGCAGCACAAGATG |
| *aacA54* | GTTAGGCCGCACAAAATCAACGCCTTATG |
| *aacA56* | GTTAGCCGGACGCTTCGCGCAGGAGTAAGAATG |
| *aacA59* | GTTAGGCAGCACAGAAGCCGCATCCCATG |
| *aacA61* | GTTAGGCAGCACAGGGCCACCGCTTATG |
| *aacA64* | GTTAGCCGGACGCCTTCGGCGCTAGGAATAAAATG |
| *aacAX* | GTTATGCATACAAATCATCACCGTGATTTACTCTTACCGGAAAGCTGAAGAAACAGATAGAGAAGCCATCTACCAAATG |
| *aacC1* | GTTAGGTGGCTCAAGTATG |
| *aacC2* | GTTAGGTGGCTCAATG |
| *aacC3* | GTTAGGCAGCAGCAGCTAAGATG |
| *aacC4* | GTTAGGTGGCTCACGTATG |
| *aacC5* | GTTAGGCATCAGGAGCAGACGAGTGTCAGTCGAAATCATCCATCTCACTGGAAACGATGTTGCGATG |
| *aacC6* | GTTAGGTGGCTCAATG |
| *aacC11* | GTTAGGTGGCTCACGTATG |
| *aacC13* | GTTAGGCATTAGGAGCCGATGAATG |
| *aadA1* | GTTAAACATCATG |
| *aadA2* | GTTAGACATCATG |
| *aadA4* | GTTAGGCATCTTCATG |
| *aadA5* | GTTAGGCATCATG |
| *aadA6* | GTTAGACATCATG |
| *aadA7* | GTTAGACATCATG |
| *aadA9* | GTTAGACATG |
| *aadA10* | GTTAGACATCATG |
| *aadA11* | GTTAGACATCATG |
| *aadA13* | GTTAGACATCATG |
| *aadA16* | GTTAGACATCATG |
| *aadA24* | GTTAGACATCATG |
| *aadA28* | GTTAGACATCATG |
| *aadA29* | GTTAGACATCATG |
| *aadA34* | GTTAGACATCATG |
| *aadB* | GTTAGGCCGCATG |
| *aphA15* | GTTAGACCGCTATG |
| *aphA16* | GTTAGCTTGACGCTCCGCGCAGGAAAGAGAAAATG |
| *blaOXA9* | GTTATGCACCTATTAAGCGCACAGCGGAGCAATG |
| *dfrA5* | GTTAACCCGGAACCAAAATTGTGAAAGTATCATTAATG |
| *fosG* | GTTATGTTTGTTAAGGTAGATTTGTGCTCCGAGGAATG |
| *aacA1* alt | GTTAGGGCGACGCCGCTATTGCGGCGCGAATACAAAGAGGAAGAGATG |
| *aacA4* alt | GTTAGGCATCACAAAGTACAGCATCATG |
| *aacA43* alt | GTTAGCCAGACGCTTCGCGCCGAGGACAATTGATG |
| *aacA47* alt | GTTATGCATCACAGAACCACCATACCTATG |
| *aacAX* alt | GTTATGCATACAAATCATCACCGTGATTTACTCTTACCGGAAAGCTGAAGAAACAGATAGAGAAGCCATCTACCAATTGTATTGCTTGGTAATG |
| *aacC5* alt | GTTAGGCATCAGGAGCAGACGAATG |
| *aadA9* alt | GTTAGACATGATG |
