# Supplementary material for: The expression of aminoglycoside resistance genes in integron cassettes is not controlled by riboswitches
Source: Nucleic Acids Res. 2022 Aug 10;50(15):8566–79. doi: 10.1093/nar/gkac662 (PMC9410878; doi:10.1093/nar/gkac662)
Supplement: gkac662_Supplemental_Files [file gkac662_supplemental_files.zip › Table S2.pdf]

Table S2. Strains and primers used in this study.

Bacterial strains			
**Number**	**Genetic background**	**Plasmids**	**Reference**
A072	MG1655	none	Laboratory strain
A249	“	pMBA	This study
A798	“	pMBA$_{5'\text{-}\mathit{aacA1}}$	This study
A439	“	pMBA$_{5'\text{-}\mathit{aacA2}}$	This study
A551	“	pMBA$_{5'\text{-}\mathit{aacA3}}$	This study
A438	“	pMBA$_{5'\text{-}\mathit{aacA4}}$	This study
A615	“	pMBA$_{5'\text{-}\mathit{aacA5}}$	This study
A424	“	pMBA$_{5'\text{-}\mathit{aacA7}}$	This study
A560	“	pMBA$_{5'\text{-}\mathit{aacA8}}$	This study
A605	“	pMBA$_{5'\text{-}\mathit{aacA16}}$	This study
A610	“	pMBA$_{5'\text{-}\mathit{aacA17}}$	This study
A425	“	pMBA$_{5'\text{-}\mathit{aacA27}}$	This study
A426	“	pMBA$_{5'\text{-}\mathit{aacA28}}$	This study
A612	“	pMBA$_{5'\text{-}\mathit{aacA29}}$	This study
A427	“	pMBA$_{5'\text{-}\mathit{aacA30}}$	This study
A428	“	pMBA$_{5'\text{-}\mathit{aacA31}}$	This study
A802	“	pMBA$_{5'\text{-}\mathit{aacA32}}$	This study
A429	“	pMBA$_{5'\text{-}\mathit{aacA34}}$	This study
A430	“	pMBA$_{5'\text{-}\mathit{aacA35}}$	This study
A437	“	pMBA$_{5'\text{-}\mathit{aacA37}}$	This study
A567	“	pMBA$_{5'\text{-}\mathit{aacA38}}$	This study
A565	“	pMBA$_{5'\text{-}\mathit{aacA39}}$	This study
A564	“	pMBA$_{5'\text{-}\mathit{aacA40}}$	This study
A502	“	pMBA$_{5'\text{-}\mathit{aacA42}}$	This study
A507	“	pMBA$_{5'\text{-}\mathit{aacA43}}$	This study
A801	“	pMBA$_{5'\text{-}\mathit{aacA44}}$	This study
A535	“	pMBA$_{5'\text{-}\mathit{aacA45}}$	This study
A808	“	pMBA$_{5'\text{-}\mathit{aacA46}}$	This study
A508	“	pMBA$_{5'\text{-}\mathit{aacA47}}$	This study
A509	“	pMBA$_{5'\text{-}\mathit{aacA48}}$	This study
A510	“	pMBA$_{5'\text{-}\mathit{aacA49}}$	This study
A534	“	pMBA$_{5'\text{-}\mathit{aacA50}}$	This study
A511	“	pMBA$_{5'\text{-}\mathit{aacA51}}$	This study
A540	“	pMBA$_{5'\text{-}\mathit{aacA52}}$	This study
A361	“	pMBA$_{5'\text{-}\mathit{aacA54}}$	This study
A512	“	pMBA$_{5'\text{-}\mathit{aacA56}}$	This study
A513	“	pMBA$_{5'\text{-}\mathit{aacA59}}$	This study
A473	“	pMBA$_{5'\text{-}\mathit{aacA61}}$	This study
A800	“	pMBA$_{5'\text{-}\mathit{aacA64}}$	This study
A549	“	pMBA$_{5'\text{-}\mathit{aacAX}}$	This study
A514	“	pMBA$_{5'\text{-}\mathit{aacC1}}$	This study
A542	“	pMBA$_{5'\text{-}\mathit{aacC2}}$	This study
A537	“	pMBA$_{5'\text{-}\mathit{aacC3}}$	This study
A799	“	pMBA$_{5'\text{-}\mathit{aacC4}}$	This study
A541	“	pMBA$_{5'\text{-}\mathit{aacC5}}$	This study
A533	“	pMBA$_{5'\text{-}\mathit{aacC6}}$	This study
A539	“	pMBA$_{5'\text{-}\mathit{aacC11}}$	This study
A606	“	pMBA$_{5'\text{-}\mathit{aacC13}}$	This study
A531	“	pMBA$_{5'\text{-}\mathit{aadA1}}$	This study
A607	“	pMBA$_{5'\text{-}\mathit{aadA2}}$	This study
A550	“	pMBA$_{5'\text{-}\mathit{aadA4}}$	This study
A530	“	pMBA$_{5'\text{-}\mathit{aadA5}}$	This study
A553	“	pMBA$_{5'\text{-}\mathit{aadA6}}$	This study
A609	“	pMBA$_{5'\text{-}\mathit{aadA7}}$	This study
A796	“	pMBA$_{5'\text{-}\mathit{aadA9}}$	This study
A608	“	pMBA$_{5'\text{-}\mathit{aadA10}}$	This study

A554	“	pMBA$_{5'-aadA11}$	This study
A558	“	pMBA$_{5'-aadA13}$	This study
A555	“	pMBA$_{5'-aadA16}$	This study
A557	“	pMBA$_{5'-aadA24}$	This study
A797	“	pMBA$_{5'-aadA28}$	This study
A556	“	pMBA$_{5'-aadA29}$	This study
A559	“	pMBA$_{5'-aadA34}$	This study
A532	“	pMBA$_{5'-aadB}$	This study
A538	“	pMBA$_{5'-aphA15}$	This study
A552	“	pMBA$_{5'-aphA16}$	This study
A563	“	pMBA$_{5'-dfrA5}$	This study
A561	“	pMBA$_{5'-fosG}$	This study
A562	“	pMBA$_{5'-blaOXA9}$	This study
A611	“	pMBA$_{Tm}$	This study
A601	“	pBGT	San Millán *et al.*, 2016
B536	“	pMBA$_{5'-aacA43alt}$	This study
B537	“	pMBA$_{5'-aacA47alt}$	This study
B538	“	pMBA$_{5'-aacAXalt}$	This study
B539	“	pMBA$_{5'-aadA9alt}$	This study
B540	“	pMBA$_{5'-aacA4alt}$	This study
A617	“	pMBA$_{5'-aacA1alt}$	This study
B628	“	pMBA$_{5'-aacC5alt}$	This study
B771	A605	pBAD-*armA*	This study
B772	A606	pBAD-*armA*	This study
B773	A607	pBAD-*armA*	This study
B774	A608	pBAD-*armA*	This study
B775	A609	pBAD-*armA*	This study
B776	A610	pBAD-*armA*	This study
B777	A611	pBAD-*armA*	This study
B778	A612	pBAD-*armA*	This study
B779	A615	pBAD-*armA*	This study
B780	A796	pBAD-*armA*	This study
B781	A797	pBAD-*armA*	This study
B782	A799	pBAD-*armA*	This study
B783	A801	pBAD-*armA*	This study
B784	A802	pBAD-*armA*	This study
B785	A249	pBAD-*armA*	This study
B786	A561	pBAD-*armA*	This study
B787	A562	pBAD-*armA*	This study
B788	A563	pBAD-*armA*	This study

Primers

Number	Name	Sequence (5´to 3´)
189	riboGFP F	ATGAGTAAAGGAGAAGAACTTTT
207	*aacA1* GFP R	AGTTCTTCTCCTTTACTCATTAGCGGCGTCGCCCTAAC
208	*aacA2* GFP R	GTTCTTCTCCTTTACTCATGACGCCTAACTTTGTTTTAGG
191	*aacA3* GFP R	AAGTTCTTCTCCTTTACTCATGAAATGGTCGCTCTGTGCT
209	*aacA4* GFP R	AGTTCTTCTCCTTTACTCATTGTGACGGAATCGTTGCTG
210	*aacA5* GFP R	AAGTTCTTCTCCTTTACTCATGCTGAAGTGTCTCCGTGCT
211	*aacA7* GFP R	AAAAGTTCTTCTCCTTTACTCATTGGTGCCTAACTTTG
195	*aacA8* GFP R	AAAAGTTCTTCTCCTTTACTCATGAGGACGGAGTTTGTGC
212	*aacA16* GFP R	AAAAGTTCTTCTCCTTTACTCATATAGGTAATGCTAGAAC
193	*aacA17* GFP R	AAAAGTTCTTCTCCTTTACTCATATAGGTAATACTAGAAC
213	*aacA27* GFP R	AAAAGTTCTTCTCCTTTACTCATGAGATGTTGATTCCGTG
214	*aacA28* GFP R	AAAAGTTCTTCTCCTTTACTCATAAAACCTCTTCGCGCGC
215	*aacA29* GFP R	AAAAGTTCTTCTCCTTTACTCATAGCCGTCTAACTTTG
216	*aacA30* GFP R	AAAAGTTCTTCTCCTTTACTCATTTAAGGTCTCCGAGTAG
217	*aacA31* GFP R	AAAAGTTCTTCTCCTTTACTCATGAGAACGGTCTTTGTGC
218	*aacA32* GFP R	AAAAGTTCTTCTCCTTTACTCATGGGACGGTCCTTTTGTG

219	*aacA34* GFP R	AAAAGTTCTTCTCCTTTACTCATAGCCTGGGCCTTCTAAC
220	*aacA35* GFP R	AAAAGTTCTTCTCCTTTACTCATAAGAAGGTGGCCCTGTG
221	*aacA37*GFP R	AAAAGTTCTTCTCCTTTACTCATAGCCGCGGTTAACTTTG
222	*aacA38* GFP R	AAAGTTCTTCTCCTTTACTCATGGTGTGACGGTGGTTATG
223	*aacA39* GFP R	AAAAGTTCTTCTCCTTTACTCATAATACCTCTTTGCGCG
224	*aacA40* GFP R	AAGTTCTTCTCCTTTACTCATGCGGAGTCTGGACTGTGCT
225	*aacA42* GFP R	AAAAGTTCTTCTCCTTTACTCATTCAAAGTCTCCGAGTAG
226	*aacA43* GFP R	AAAAGTTCTTCTCCTTTACTCATTTGTCCTCGGCGCGAA
227	*aacA44* GFP R	AAAAGTTCTTCTCCTTTACTCATTTAATCACCAATTGTGCTCAGTC
228	*aacA45* GFP R	AAAAGTTCTTCTCCTTTACTCATTGGCTGAGCCTTTTAAC
229	*aacA46* GFP R	AAAAGTTCTTCTCCTTTACTCATAAGATCCTCTTTCTTCACGC
230	*aacA47* GFP R	AAAAGTTCTTCTCCTTTACTCATAACTTTGTTTTAGGGCG
231	*aacA48* GFP R	AAAAGTTCTTCTCCTTTACTCATGCTCGGTGCAGGTTTAG
232	*aacA49* GFP R	AAAAGTTCTTCTCCTTTACTCATTGAAACTCTCCTCTGCG
233	*aacA50* GFP R	AAAGTTCTTCTCCTTTACTCATGAGAATTGTCTTTGTGC
234	*aacA51* GFP R	AAAAGTTCTTCTCCTTTACTCATACTGGGACGGTTCCTTG
235	*aacA52* GFP R	AAAAGTTCTTCTCCTTTACTCATCTTGTGCTGCCTAACTT
236	*aacA54* GFP R	AAAGTTCTTCTCCTTTACTCATAAGGCGTTGATTTTGTGC
237	*aacA56* GFP R	AAAAGTTCTTCTCCTTTACTCATTCTTACTCCTGCGCGAA
238	*aacA59* GFP R	AAAAGTTCTTCTCCTTTACTCATGGGATGCGGCTTCTGT
239	*aacA61* GFP R	AAAAGTTCTTCTCCTTTACTCATAAGCGGTGGCCCTGTGC
240	*aacA64* GFP R	AAAAGTTCTTCTCCTTTACTCATTTTATTCCTAGCGCCGA
241	*aacAX* GFP R	AAAAGTTCTTCTCCTTTACTCATTTGGTAGATGGCTTCTC
192	*aacC1*C4 GFPR	AAAAGTTCTTCTCCTTTACTCATACTTGAGCCACCTAACT
242	*aacC2* GFP R	AAAAGTTCTTCTCCTTTACTCATTGAGCCACCTAACTTTG
243	*aacC3* GFP R	AAAAGTTCTTCTCCTTTACTCATCTTAGCTGCTGCTGCCT
244	*aacC5* GFP R	AAAAGTTCTTCTCCTTTACTCATCGCAACATCGTTTCCAG
245	*aacC6* GFP R	AAAAGTTCTTCTCCTTTACTCATTGAGCCACCTAACTTTG
246	*aacC11* GFP R	AAAAGTTCTTCTCCTTTACTCATACGTGAGCCACCTAAC
247	*aacC13* GFP R	AAAAGTTCTTCTCCTTTACTCATTCATCGGCTCCTAATGC
248	*aadA1* GFP R	TTCTTCTCCTTTACTCATGATGTTTAACTTTGTTTTAGGGC
190	*aadA2 6 7 10 11 13 16 24 28 29 34* GFP B R	CTAAAACAAAGTTAGACATCATGAGTAAAGGAGAAGAACT
249	*aadA4* GFP R	AAAAGTTCTTCTCCTTTACTCATGAAGATGCCTAACTTTG
250	*aadA5* GFP R	AAAAGTTCTTCTCCTTTACTCATGATGCCTAACTTTG
251	*aadA9* GFP R	AAGTTCTTCTCCTTTACTCATGTCTAACTTTGTTTTAGGGCG
252	*aadB* GFP R	AAAAGTTCTTCTCCTTTACTCATGCGGCCTAACTTTGTTT
253	*aphA15* GFP R	AAAAGTTCTTCTCCTTTACTCATAGCGGTCTAACTTTGTT
254	*aphA16* GFP R	AAAAGTTCTTCTCCTTTACTCATTTTCTCTTTCCTGCGCG
454	*dfrA5* GFP R	GTTCTTCTCCTTTACTCATTAATGATACTTTCACAATTTTGGTTCC
455	*fosG* GFP R	AGTTCTTCTC CTTTACTCAT TCCTCGGAGCACAAATCTAC
453	*blaOXA9* GFP R	AGTTCTTCTCCTTTACTCATTGCTCCGCTGTGCGCTTAAT
450	Tm F	AGTCGCCCTAAAACAAAGTTTCGATTTTAAGATAACTTTACGTG
449	Tm R	AAAAGTTCTTCTCCTTTACTCATTGCGTTCTACTCTGAAG
759	*aacA1* alt R	CTCTTTGTATTCGCGCCGCAATAGCGGCGTCGCCC
760	*aacA43* alt R	GTTCTTCTCCTTTACTCATCAATTGTCCTCGGCGC
761	*aacA4* alt R	TCTTCTCCTTTACTCATGATGCTGTACTTTGTGATGC
762	*aadA9* alt R	TCTCCTTTACTCATCATGTCTAACTTTGTTTTAGGGC
763	*aacA47*alt R	GTTCTTCTCCTTTACTCATAGGTATGGTGGTTCTGTG
764	*aacAX* alt R	TTCTTCTCCTTTACTCATTACCAAGCAATACAATTGGTAG
837	*aacC5* alt R	TTCTCCTTTACTCATTCGTCTGCTCCTGATGCC
